# *De novo* transcriptome assembly and functional analysis reveal a dihydrochalcone 3-hydroxylase(DHC3H) of wild *Malus* species that produces sieboldin *in vivo*

**DOI:** 10.3389/fpls.2022.1072765

**Published:** 2022-12-16

**Authors:** Simón Miranda, Jorge Lagrèze, Anne-Sophie Knoll, Andrea Angeli, Richard V. Espley, Andrew P. Dare, Mickael Malnoy, Stefan Martens

**Affiliations:** ^1^ Research and Innovation Center, Fondazione Edmund Mach, San Michele all’Adige, Trento, Italy; ^2^ Center Agriculture Food and Environment (C3A), University of Trento, Trento, Italy; ^3^ The New Zealand Institute for Plant and Food Research Limited, Auckland, New Zealand

**Keywords:** dihydrochalcones, 3-hydroxyphloretin, polyphenols, biosynthesis, cytochrome P450, 3-hydroxylation, *Malus* species

## Abstract

Sieboldin is a specialised secondary metabolite of the group of dihydrochalcones (DHC), found in high concentrations only in some wild *Malus* species, closely related to the domesticated apple (*Malus* × *domestica* L.). To date, the first committed step towards the biosynthesis of sieboldin remains unknown. In this study, we combined transcriptomic analysis and a *de novo* transcriptome assembly to identify two putative 3-hydroxylases in two wild *Malus* species (*Malus toringo* (K. Koch) Carriere syn. *sieboldii* Rehder*, Malus micromalus* Makino) whose DHC profile is dominated by sieboldin. We assessed the *in vivo* activity of putative candidates to produce 3-hydroxyphloretin and sieboldin by *de novo* production in *Saccharomyces cerevisiae*. We found that CYP98A proteins of wild *Malus* accessions (CYP98A195, *M. toringo* and CYP98A196, *M. micromalus*) were able to produce 3-hydroxyphloretin, ultimately leading to sieboldin accumulation by co-expression with PGT2. CYP98A197-198 genes of *M.* × *domestica*, however, were unable to hydroxylate phloretin *in vivo*. CYP98A195-196 proteins exerting 3-hydroxylase activity co-localised with an endoplasmic reticulum marker. CYP98A protein model from wild accessions showed mutations in key residues close to the ligand pocket predicted using phloretin for protein docking modelling. These mutations are located within known substrate recognition sites of cytochrome P450s, which could explain the acceptance of phloretin in CYP98A protein of wild accessions. Screening a *Malus* germplasm collection by HRM marker analysis for CYP98A genes identified three clusters that correspond to the alleles of domesticated and wild species. Moreover, CYP98A isoforms identified in *M. toringo* and *M. micromalus* correlate with the accumulation of sieboldin in other wild and hybrid *Malus* genotypes. Taken together, we provide the first evidence of an enzyme producing sieboldin *in vivo* that could be involved in the key hydroxylation step towards the synthesis of sieboldin in *Malus* species.

## Introduction

Dihydrochalcones (DHCs) are specialised metabolites with a limited natural distribution, being isolated from some medicinal plants but abundantly accumulated in *Malus* species (Rivière, 2016). DHCs are categorised as minor flavonoid together with flavanones, flavanonols, chalcones and retrochalcones because of their restricted natural occurrence (Tomás-Barberán and Clifford, 2000). In 1835, the first DHC, phloridzin (phloretin 2′-*O*-glucoside), was described as an antipyretic substance isolated from bark and root of cultivated apple tree (Gosch et al., 2010b). For a long time, phloridzin was believed to be exclusive to *Malus* × *domestica* L, where it can account for up to 10 – 14% of leaf dry weight (Gosch et al., 2010b). Thus, phloridzin has been used as an effective marker to detect food-derived adulteration with apple (Spinelli et al., 2016; Hrubá et al., 2021). However, trilobatin and sieboldin dominate the polyphenolic profile of some wild *Malus* relatives (Gutierrez et al., 2018b). DHCs have demonstrated a wide range of beneficial effects against cancer, cardiovascular and free radical-involving diseases (Stangl et al., 2005; Dugé de Bernonville et al., 2010; Szliszka et al., 2010; Viet et al., 2013). Some DHCs have also been reported to act as flavour sweeteners and bitterness blockers, such as trilobatin and neohesperidin DHC (Tomás-Barberán et al., 1995; Wang et al., 2020). According to the currently proposed DHC pathway, 3-hydroxyphloretin, the key precursor of sieboldin in *Malus* species, would also be an intermediate compound towards the biosynthesis of the flavour enhancer neohesperidin DHC (Ibdah et al., 2018). Phloridzin may act as an anti-diabetic compound by blocking sodium-linked glucose transport and renal reabsorption of glucose in kidneys (Ehrenkranz et al., 2005). Despite the protective effects reported in mammal models, little is known about how these metabolites are biosynthesised and what is their function *in planta*.

Previous studies have suggested a role of DHCs in plant defence during *Erwinia amylovora* and *Venturia inaequalis* infection, pathogens causing fire blight and scab disease in apples, respectively. Although the relevance of phloridzin and its aglycone precursor phloretin in the acquisition of pathogen resistance is still controversial, it has been proposed that DHCs accumulate to cope with the oxidative stress during pathogen response (reviewed in Gosch et al., 2010). Moreover, phloridzin and its aglycone precursor phloretin have shown antibacterial and antifungal properties (Lattanzio et al., 2001; Oleszek et al., 2019; Liu et al., 2021). In addition to the most studied DHCs, sieboldin exhibits antibacterial and antifungal activities, suggesting a complementary role to phloretin and phloridzin against *E. amylovora* and *V. inaequalis* infection (Gaucher et al., 2013). Furthermore, overexpressing a chalcone 3-hydroxylase from *Cosmos sulphureus* Cav. in apple led to increased levels of 3-hydroxyphloridzin, correlating with reduced susceptibility to apple scab and fire blight (Hutabarat et al., 2016). Along with the roles of DHCs in biotic stresses, it has been reported that sieboldin could be involved in the tolerance to oxidative stress in *Malus* leaves (Dugé de Bernonville et al., 2010).

To get a better understanding of the accumulation of DHCs in plants, a number of studies have attempted to elucidate the biosynthetic pathway, providing a valuable resource to assess their physiological roles. The DHC pathway (**Supplementary Figure 1**) diverts from the main phenylpropanoid pathway from *p*-coumaroyl-CoA by the action of a postulated double bond reductase. Then, chalcone synthase (CHS) catalyses the condensation of *p*-dihydrocoumaroyl-CoA to phloretin (Gosch et al., 2010b). Phloretin can be directly glycosylated at position 2′- or 4′ by Ph-2′ or Ph-4′-*O*-UDP-glycosyltransferases (**Supplementary Figure 1**) to produce phloridzin or trilobatin, respectively. Several studies have identified 2′-*O*-UDP-glycosyltransferases involved in the synthesis of phloridzin (PGT1, Jugdé et al., 2008; Gosch et al., 2010a; Gosch et al., 2012; Yahyaa et al., 2016; Zhou et al., 2017). However, the enzyme responsible for the 4′-*O*-glycosylation of trilobatin was characterised only recently (PGT2, Wang et al., 2020). Sieboldin has been postulated to derive from hydroxylation of phloretin in position 3 of B-ring before being glycosylated (Ibdah et al., 2018; **Supplementary Figure 1**). Although the glycosylation of 3-hydroxyphloretin can be carried out at least by PGT2 to produce sieboldin *in vitro* (Wang et al., 2020), the first committed step to 3-hydroxyphloretin has not been yet identified. To date, the only attempt to identify genes involved in this step resulted in the characterisation of two flavonoid 3′-hydroxylases from *M.* × *domestica* (***F3*** *′* ***HI*** and ***F3*** *′* ***HII***), which did not accept phloretin and therefore unlikely to be involved in its 3-hydroxylation (Weissensteiner et al., 2021). In this study, we aimed to identify candidate genes that may account for the 3-hydroxylation of phloretin, that could be involved in DHC formation of wild *Malus* species. Combining transcriptomic, metabolic and functional analyses, our work reports for the first time a 3-hydroxylase of *Malus* species that convert phloretin to 3-hydroxyphloretin *in vivo*, the key missing step towards the production of the specialised metabolite sieboldin naturally occurring in some *Malus* species.

## Materials and methods

### Plant material

Plants of the *Malus* germplasm collection of Fondazione Edmund Mach in Trento, Italy (GPS coordinates 46.181848, 11.119849), consisting in three individuals per accession (**Supplementary Table 1**) planted in 2011 on M9 T337 rootstock and maintained at the germplasm collection, with winter pruning, spring low-titre nitrogen-based fertilisation and phytosanitary treatments during the growing season following the integrated production regulations of the Autonomous Province of Trento were used in this study. RNA-Seq, *de novo* transcriptome assembly and functional analysis was carried out on three individuals of cultivated apple (*M.* × *domestica* cv. ‘Golden Delicious’) and two wild *Malus* species (*M. toringo* syn. *sieboldii*, PI 613858; *M. micromalus*, PI 594092) Samples were collected from young and old leaves, and five time points of bud development (dormant, half inch, tight cluster, pink, bloom). Fruits were collected at different time points representing the main physiological changes described during fruit development (Janssen et al., 2008): fruit set, and fruit at 35, 60, 87 and 132 days after full bloom (DAFB). At ripening, fruit were split in two enriched fractions of skin and pulp. Sampling was conducted in the three plants representing each accession in the germplasm collection, and each sample comprised at least 10 fruit per time point. All samples were immediately frozen in liquid nitrogen and stored at -80°C until further processing for molecular and metabolic analysis.

### RNA extraction, library construction and RNA-Seq analysis

Total RNA was isolated from 100 mg of fresh frozen sample by CTAB extraction and LiCl precipitation, followed by cleaning using Spectrum™ Plant Total RNA kit (Sigma-Aldrich). RNA quality and quantity were assessed before and after DNase treatment by gel electrophoresis and spectrophotometric measurements of absorbance ratios at 260 and 280 (A260/280) or 230 nm (A260/230). RNA samples with A260/280 and A260/230 ratios between 1.8 - 2.0 were considered acceptable and used for downstream applications. We treated 5 µg of total RNA with TURBO™ DNase (Invitrogen). RNA integrity was determined by Agilent 2100 Bioanalyzer, and RIN values > 8.0 were further used. Three cDNA libraries per accession were prepared from total RNA of young leaves, using random primers and the TrueSeq^®^ stranded Illumina kit and then sequenced by HiSeq 2500 Illumina sequencing system to obtain single-stranded 100 bp reads. Quality filtering and adapter trimming was performed by *fastp*, with a minimum length set at 50 bp. Then, filtered reads were aligned to *M.* × *domestica* genome GDDH13 (Daccord et al., 2017) and counted using featureCounts (Liao et al., 2014). Differentially expressed genes (DEGs) were identified by DESeq2 (Love et al., 2014) in R-project environment, and *p-*values were adjusted using the procedure of Benjamini-Hochberg. Genes with |Log_2_(Fold change)| > 2 and *p-adj* < 0.05 were considered as DEGs. Enrichment analysis with Kyoto Encyclopedia of Genes and Genomes (KEGG) was performed by KOBAS v3.0 (http://kobas.cbi.pku.edu.cn/kobas3/).

### *De novo* transcriptome assembly and functional annotation

*De novo* transcriptome assemblies of the two wild *Malus* spp. (*M. toringo*, *M. micromalus*) were assembled using Trinity software v 2.9.1 using three biological replicates per accession, with a default k-mer size of 25 and minimum contig length of 200. To remove redundant and alternatively spliced transcripts, identical and near-identical contigs were clustered by CD-HIT-EST tool v4.6, with a similarity threshold of 95%. The quality of the assemblies was evaluated by mapping with Bowtie 2 v2.3.2 to compute contig ExN50 statistics. Transcriptome completeness was assessed by BUSCO v5.2.2. Gene open reading frames (ORFs) were predicted by TransDecoder v5.5.0 using the assembled unique transcripts as input. Functional annotation of the assembled transcripts was conducted using Diamond software v2.0.8.0 by protein BLAST against the non-redundant (*nr*) database of NCBI with an E-value cut-off of 10^-5^. Orthologs to *M.* × *domestica* were predicted by a reciprocal-best hit approach (RBH), with 70% minimum percentage of identity and 50% minimum percentage query coverage.

### Gene expression analysis

We used 2 µg of DNase-treated RNA to synthesise single-stranded cDNA using SuperScript™ III Reverse Transcriptase (Invitrogen) and oligo(dT)_20_ following manufacturer’s instructions. Gene-specific primer sets (**Supplementary Table 2**) were designed by Sigma OligoArchitecht. Quantitative RT-PCR (RT-qPCR) reactions were run in a C1000 Touch thermal cycler coupled to CFX96 Detection System (Bio-Rad) using 2X SsoFast™ EvaGreen^®^ Supermix (Bio-Rad). Reactions consisted of 10 ng cDNA, 250 nM of forward and reverse primers in a total volume of 12.5 µL, and were run with a standard thermal profile: 98°C, 5 s; 40 cycles of 98°C, 5 s, 60°C, 10 s, 72°C, 10 s; with a final melting step between 65°C and 95°C. Primer efficiency was determined by LinRegPCR v. 7.5 from amplification plots. The quality of PCR reactions was determined by analysis of the dissociation and amplification curves. The constitutively expressed genes *ACT2* (actin 2), *EF1α* (elongation factor 1 alpha) and *UBI* (ubiquitin 10) were tested as candidate normalizers. Transcript levels of target genes were conducted in three biological replicates and normalised to the expression of *EF1α*, which was the most stably expressed gene under the tested conditions according to RefFinder (https://www.heartcure.com.au/reffinder/).

### Targeted metabolomic profiling

Young leaves of each accession in the *Malus* germplasm collection and samples from leaves, buds, flowers, and fruits of *M. toringo*, *M. micromalus* and *M.* × *domestica* cv. ‘Golden Delicious’ were ground and 100 mg fresh weight (FW) was extracted in 5 mL 80% v/v methanol, sonicated for 20 min at 60 Hz in a water bath at 25°C and kept in dark for 48 h, and then filtered through a 0.22 µm PTFE filter and stored at 4°C. Phenolic compounds from yeast cultures were extracted by ethyl acetate. Briefly, 1 mL of culture of three independent clones was extracted twice with 1 volume ethyl acetate followed by centrifugation at 16,000 *g* for 2 min. The supernatants were collected in 2 mL microcentrifuge tubes and evaporated in an Eppendorf Concentrator Plus ™, by incubation at 25°C for 1 h under negative pressure. Pellets were resuspended in 200 µL 80% v/v methanol and stored at 4°C until further analysis. Targeted profiling of phenolic compounds was conducted using a Waters Acquity UPLC system coupled to a Waters Xevo^®^ TQ-MS mass spectrometer equipped with an electrospray source using multiple reaction monitoring (MRM), as previously described (Vrhovsek et al., 2012). Summary with MRM parameters used for each metabolite is reported in **Supplementary Table 3**. Data processing was carried out with default settings in Mass Lynx™ and TargetLynx™ softwares (Waters Co., USA). Metabolite concentration automatically determined by TargetLynx™ software using calibration curves of real metabolite standards was normalised according to the fresh weight of plant material (mg/mg FW), or culture volume used for extraction corrected by the dilution factor after resuspension in methanol (mg/L).

### Metabolic engineering of *Saccharomyces cerevisiae*

We used a *S. cerevisiae* strain derived from S288C (*MATα hoΔ0 his3Δ0 leu2Δ0 ura3Δ0)*, which produces phloretin by simultaneous expression of *HaCHS*, *ScTSC13*, *At4CL2*, *AtPAL2*, *AmC4H*, *ScCPR1* in a vector conferring prototrophy to uracil (*PAR1*; Eichenberger et al., 2017). To attempt *in vivo* conversion of phloretin into 3-hydroxyphloretin in *S. cerevisiae*, coding sequences of putative 3-hydroxylases were cloned into MCS-2 of pAT425 yeast expression vector (Ishii et al., 2014) by adding *SalI* and *NotI* restriction recognition sites through PCR using Phusion Hot Start II DNA Polymerase (ThermoFisher), followed by digestion with FastDigest™ SalI and NotI (ThermoFisher) and ligation using T4 DNA ligase (ThermoFisher), according to manufacturer’s instructions. To achieve conversion of phloretin into sieboldin, the coding sequence of *MtorPGT2*-2 (GenBank accession number MN381000) was cloned by PCR amplification with primers incorporating *FseI* and *AvrII* restriction sites, and digested with FseI and AvrII (New England Biolabs) to be ligated into the MCS-1 of pAT425 vector harbouring putative 3-hydroxylases. Ligation reactions were transformed into chemically competent *E. coli* OneShot™ Mach1™ cells (ThermoFisher) and plated in LB agar plates (5 g/L yeast extract, 10 g/L tryptone, 5 g/L NaCl, 15 g/L agar) supplemented with carbenicillin 100 µg/mL. Confirmation of correct cloning was assessed by Sanger sequencing. Transformation of *PAR1* strain was conducted by a standard lithium acetate method (Gietz and Schiestl, 2007), plated in minimal SC dropout medium without uracil and leucine (SC-U-L) and grown at 30°C for 3 days. Positive clones were inoculated into 50 mL of SC-U-L and incubated at 30°C, 250 rpm for 72 – 96 h. Final OD600 of a 1:10 dilution was measured and 1 mL of three independent clones per construct was sampled for metabolite profiling.

### Subcellular localization

Tobacco (*Nicotiana tabacum* L. cv. ‘Samsun-NN’) was stably transformed with fluorescent markers of plasma membrane (pm-rk, CD3-1007, ABRC) and endoplasmic reticulum (ER-rk, CD3-959, ABRC) fused to mCherry, as previously described (Japelaghi et al., 2018). CYP98A196 was amplified by PCR using specific primers (**Supplementary Table 2**), cloned into pCR8™/GW/TOPO™ (Invitrogen) and recombined into pEarleyGate 101 (Earley et al., 2006) by Gateway™ LR™ clonase enzyme mix (Invitrogen) according to manufacturer instructions to produce a C-terminal protein fusion to EYFP. Tobacco leaves were agroinfiltrated following a standard protocol for transient expression (Sparkes et al., 2006) with *A. tumefaciens* C58C1 (pMP90) harbouring the empty vector pEarleyGate101 or the construct CYP98A196-EYFP. Tobacco leaf protoplasts were prepared following the protocol of Hsu et al., 2021. Confocal imaging was performed 72 hours after agroinfiltration and 16 h after protoplast digestion, using a Leica TCS SP8 confocal microscope and Leica Application Suite X software. EYFP and mCherry signals were visualised with excitation at 488 and 554 nm, respectively. Emission was collected between 510 to 540 nm and 590 to 650 nm for EYFP and mCherry. Autofluorescence of chlorophyll was detected between 660 to 730 nm. Z-stacking was performed in 1 to 5 µm steps, in sections ranging 10 to 50 µm. Image processing consisted of merging acquisition channels and quantifying colocalization.

### High resolution melting analysis

DNA was extracted from *Malus* accessions in the germplasm collection of Fondazione Edmund Mach, as previously described (Allen et al., 2006). HRM analysis was performed in a C1000 Touch thermal cycler coupled to CFX96 Detection System (Bio-Rad) using 2X SsoFast™ EvaGreen^®^ Supermix (Bio-Rad). Reactions consisted of 10 ng genomic DNA, 500 nM of specific primers (**Supplementary Table 2**) in a total volume of 20 µL, and were run with the following thermal profile: 98°C, 2 min; 40 cycles of 98°C, 5 s, 60°C, 10 s; with a melting step between 60°C to 95°C, with acquisitions in 0.1°C inc./sec., 5 sec./step. Normalisation and temperature shift determination of melting curves were determined with default settings in the Precision Melt Analysis™ software (Bio-Rad). Clusters were automatically calculated using a default Tm difference threshold of 0.15 degrees and pre-melt and post-melt regions were optimised to 70.9 - 71.4°C and 78.3 - 78.8°C, respectively.

### Protein modelling and phylogenetic analysis

Protein models were obtained using a plant P450 database (PCPD, http://p450.biodesign.ac.cn/). Protein docking was performed using the predicted protein structures obtained in PCPD and phloretin as a ligand in the web-based tool PCPLD.

Publicly available protein sequences of F3′H, F3′5′H and F6H, as well as CYP98A family members were used to build phylogenetic trees with candidate proteins from *M. toringo* and *M. micromalus* using MEGA11. Phylogenetic tree was inferred by the maximum likelihood method with 1000 bootstrap replicates and JTT matrix-based model.

### Statistical analysis and data visualisation

Statistical differences among tissues and genotypes for metabolite and expression analysis were determined by Statistica software using two-way analysis of variance (ANOVA), with Tukey *post-hoc* HSD test, using *p* value < 0.05 as statistically significant. Results were displayed in GraphPad Prism v6.0.

## Results

### Targeted phenolic metabolomic profiling of domesticated apple (*M.* × *domestica*), *M. toringo* and *M. micromalus* wild accessions

**Figure 1:**
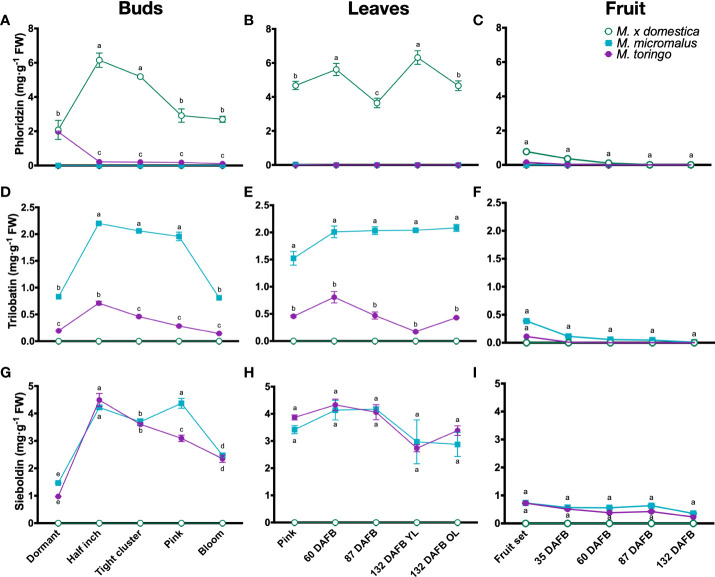
Metabolic profiling of DHCs in wild *Malus* and domesticated apple. Content of phloridzin **(A–C)**, trilobatin **(D–F)** and sieboldin **(G-I)** during bud **(A, D, G)**, young (YL) and old (OL) leaf **(B, E, H)** and fruit **(C, F, I)** development of *M. toringo*, *M. micromalus* and *M.* × *domestica* cv. ‘Golden Delicious’. Fruits were sampled at fruit set and 60, 87, 132 days after full bloom (DAFB). Values are reported in mg·g^-1^ fresh weight (FW), and represent the mean ± SE of three biological replicates in each sampling point, corresponding to the three individuals representing each accession in the *Malus* germplasm collection. Letters (a-d) indicate statistical differences (*p* < 0.05) across genotypes and sampling points as determined by two-way ANOVA and Tukey’s HSD test.

**Figure 2:**
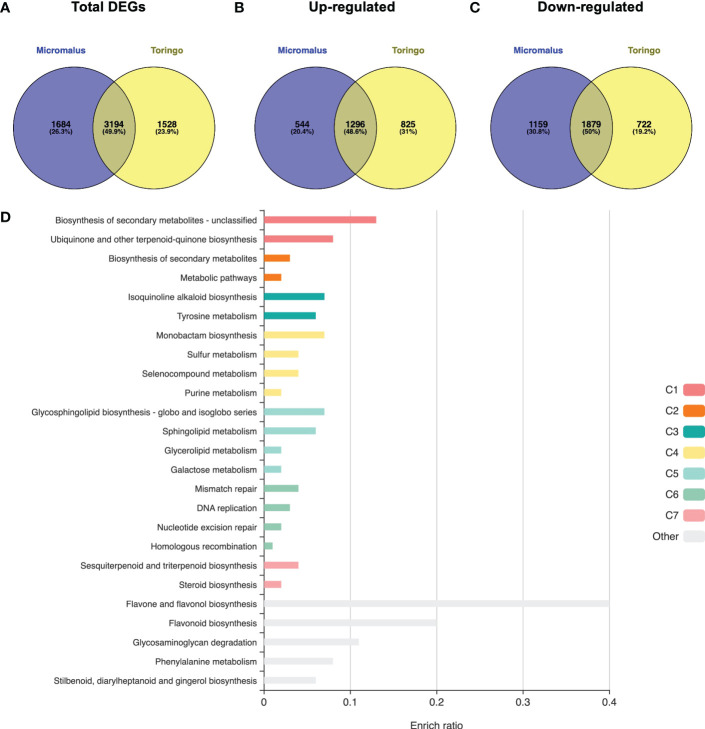
Transcriptomic overview of RNA-Seq results for wild *Malus* accessions compared to domesticated apple. Venn diagram of total number of DEGs **(A)**, up-regulated **(B)** and down-regulated **(C)** DEGs of each *Malus* genotype. **(D)** Overrepresentation analysis identified statistically significant KEGG pathways enriched in the common list of up- and down-regulated DEGs of wild *Malus* species, compared to domesticated apple. Bar size represents the enriched ratio calculated as the number of genes included in the list compared to the background reference *Malus* × *domestica* genome.

**Figure 3:**
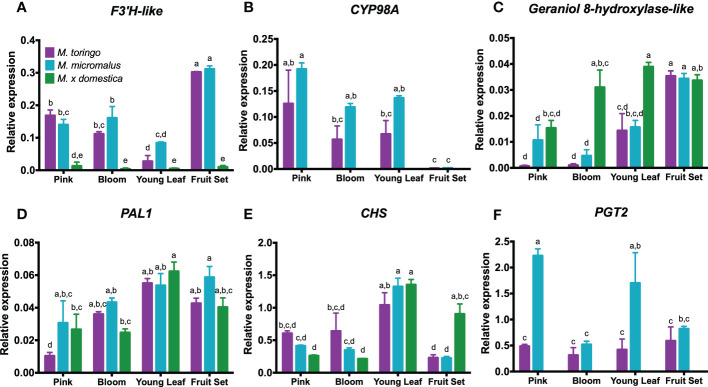
Transcriptional regulation of candidate 3-hydroxylase genes from wild *Malus* accessions and genes involved in the phenylpropanoid pathway. The expression of *F3′H-like* **(A)**, *CYP98A* **(B)** and geraniol 8-hydroxylase-like **(C)** genes was assessed in blooming and pink flowers, young leaves and fruit set of *M. toringo*, *M. micromalus* and *M.* × *domestica* cv. ‘Golden Delicious’ by RT-qPCR. In addition, phenylalanine ammonia lyase (*PAL1*), chalcone synthase (*CHS*) and 4′-*O*-glycosyltransferase (*PGT2*) genes were assessed. Bars represent mean ± SE for three biological replicates. Letters (a-e) indicate statistical differences (*p* < 0.05) across genotypes and sampling points as determined by two-way ANOVA and Tukey’s HSD test.

**Figure 4:**
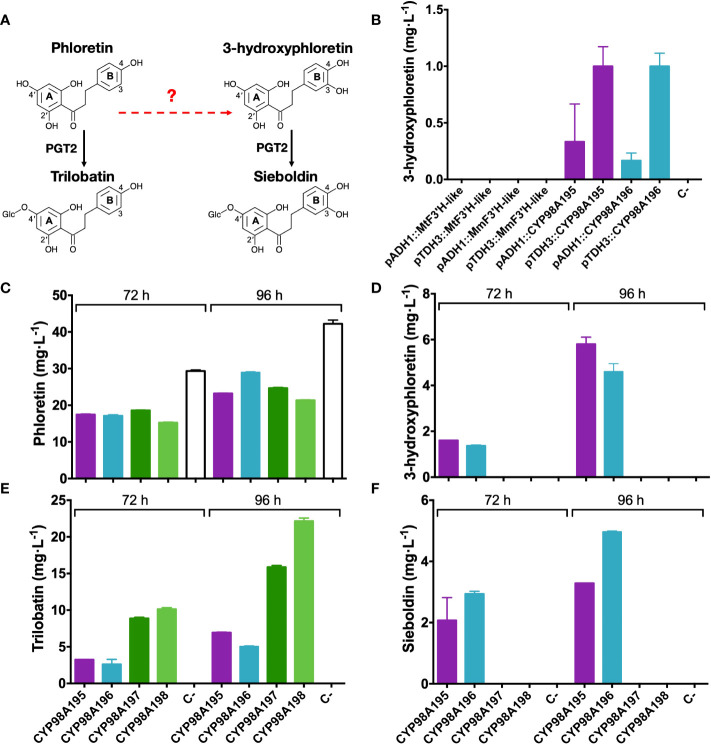
Functional characterisation of 3-hydroxylase candidate genes from wild and domesticated *Malus* species. **(A)** Simplified overview of the phenylpropanoid pathway reconstructed in *S. cerevisiae*, where the characterised enzymatic steps are indicated by solid arrows and 3-hydroxylation reaction potentially catalysed by candidate genes tested is indicated by a red dashed arrow. Relevant carbon residues are indicated by numbers in ring A (2*′* and 4*′*) and B (3 and 4) of DHCs. **(B)** Detection of 3-hydroxyphloretin after 48 h culture from yeast expressing *F3′H-like* or *CYP98195 and CYP98A196* genes of *M. toringo*, *M. micromalus*, respectively or *M.* × *domestica* (*CYP98A197*, *CYP98A198*) under the control of ADH1 or TDH3 constitutive promoters. In addition, phloretin **(C)**, 3-hydroxyphloretin **(D)**, trilobatin **(E)** and sieboldin **(F)** were detected by a targeted UPLC/QqQ/MRM method for phenolic compounds, after incubation for 72 and 96 h of a phloretin-producing *S. cerevisiae* co-expressing *PGT2* and *CYP98A* genes of wild and domesticated *Malus* species. Values represent the mean ± SE of extractions from three independent yeast cultures. Negative control (C-) corresponds to the phloretin-producing strain transformed with the empty pAT425 vector.

To get a detailed profiling of phenylpropanoids throughout the development of wild and domesticated *Malus* species, we sampled plants of *M. toringo*, *M. micromalus* and *M.* × *domestica* cv. ‘Golden Delicious’, which are represented by three individuals in the germplasm collection of Fondazione Edmund Mach (Italy). Plants were sampled at different bud stages (dormant, half inch, tight cluster, pink and bloom), and across fruit development (fruit set and 35, 60, 87 and 132 DAFB). Young leaves (YL) were collected in the same time points, along with old leaves (OL) at 132 DAFB of fruit development (**Supplementary Figure 2**). We examined compounds of the DHC pathway based on a targeted metabolomic profiling of phenolic compounds (Vrhovsek et al., 2012). LC-MS/MS analysis shows that DHC profiles in all tissues of wild accessions are dominated by sieboldin and trilobatin (**Figures 1D-I**), whilst phloridzin is present only in the domesticated apple (**Figures 1A-C**). Sieboldin is the predominant DHC found in leaves and bud development of wild species, ranging from 3 to 4 mg/g FW in leaves (**Figure 1H**) and 1 to 4 mg/g FW in buds (**Figure 1G**). Meanwhile, trilobatin content varies from 0.1 to 2 mg/g FW in buds and leaves (**Figures 1D, E**), showing significant higher abundance in *M. micromalus* compared to *M. toringo*. In buds, trilobatin and sieboldin show a sharp significant increase from dormant to half inch buds, which is maintained until pink flowers, when both DHCs decrease at bloom stage (**Figures 1D, G**). In leaves, trilobatin and sieboldin content show no statistical difference during the growing season, tending to be stable across the season (**Figures 1E, H**). The content of sieboldin and trilobatin in whole fruits is 2.5- to 10-fold and 2- to 20-fold lower compared to leaves, respectively, and shows no statistical difference throughout fruit development (**Figures 1F, I**). Moreover, skin-enriched fraction accounts for the majority of phloridzin, trilobatin and sieboldin in fruits, in both domesticated and wild species (**Supplementary Figures 3G-I**). In addition, we detected the aglycone phloretin in leaves and buds of all accessions (**Supplementary Figures 3D-F**), whilst 3-hydroxyphloretin was predominantly found in leaves, followed by buds and fruit set (**Supplementary Figures 3A-C**).

### RNA-Seq and *de novo* assembly of leaf transcriptome of *M. toringo* and *M. micromalus*

The transcriptomes of young leaves of *M. toringo* and *M. micromalus* were sequenced, as well as the *M.* × *domestica* cv. ‘Golden Delicious’. A total of 272,391,814 high-quality 100-bp reads were obtained, with an average of 90,8 million reads per genotype (**Supplementary Table 4**). GC content of all accessions was 47 – 48%. Between 97.6 – 97.7% and 96.2 – 96.3% bases showed quality scores of Q20 and Q30, respectively. Following adapter trimming and quality filtering, ~98.5% and ~97.1% bases showed Q20 and Q30 scores, indicating that the quality of filtered reads was adequate for further analysis. Aiming to identify differentially expressed genes (DEGs) in wild *Malus* species compared to domesticated apple, we firstly conducted an RNA-Seq analysis by mapping clean reads by STAR to *M.* × *domestica* reference genome GDDH13 (Daccord et al., 2017). Transcriptomes of wild species mapped 71.5% (*M. micromalus*) and 73.01% (*M. toringo*) to *M.* × *domestica* ‘Golden delicious’ genome, which was similar to the mapping of reads from ‘Golden Delicious’ (72.7%, **Supplementary Table 4**), whereas unassigned reads due to multiple or ambiguous mapping ranged 24 – 26% and 0.12 – 0.13%, respectively. Mapped reads were counted by featureCounts and identification of DEGs was conducted by DESeq2, considering |fold change| > 2 and *p-adj* < 0.05. Among the 4,878 and 4,722 genes considered as DEGs in *M. micromalus* and *M. toringo* using apple ‘Golden delicious’ reference genome (**Supplementary Table 5**), we found a common core of 1296 up- and 1,879 down-regulated genes (**Figures 2A-C**). Gene set enrichment (GSE) analysis using KEGG pathways identified statistically enriched terms in this common set of DEGs, including ‘biosynthesis of secondary metabolites’, ‘ubiquinone and other terpenoid-quinone biosynthesis’ and ‘tyrosine metabolism’ (**Figure 2D**). Since sieboldin is highly accumulated in *M. micromalus* and *M. toringo* leaves, we hypothesised that potential genes involved in its biosynthesis would be among up-regulated DEGs. In order to get the actual coding sequences of up-regulated genes of *M. micromalus* and *M. toringo*, we performed a *de novo* transcriptome assembly. The summary of results is presented in **Table 1**. Trinity (Grabherr et al., 2013) assembled 139,716 and 132,885 transcripts for *M. toringo* and *M. micromalus*, respectively. The expression-informed ExN50 statistic was used as previously described (Hölzer and Marz, 2019), resulting in a transcript length of 2,636 and 2,585 bp in *M. toringo* and *M. micromalus*. For each accession, a total of 104,138 and 97,838 unigenes using CD-HIT-EST, and 38,730 and 36,443 open reading frames (ORFs) by TransDecoder were identified. We further evaluated the quality of *de novo* transcriptome assemblies using BUSCO, a tool that assesses the completeness of transcriptomes by finding a set of near-universal single copy orthologs (Simão et al., 2015). Transcriptomes of *Malus* species showed 90% of complete (unique and duplicated) ORFs (**Table 1**). In addition, we used the RBH approach to identify orthologs of *M.* × *domestica* ‘Golden Delicious’ in wild accessions, which led to 13,860 and 13,734 orthologs in *M. toringo* and *M. micromalus*, respectively. CDS obtained by TransDecoder were aligned against the non-redundant (Nr) database of NCBI, to provide functional annotation that could serve as the basis for retrieving candidate genes to be further analysed (**Supplementary Table 6**). Combining RNA-Seq results, *de novo* transcriptomes and functional annotation, we identified two potential 3-hydroxylases for further analyses.

**Table 1:** Statistics of *de novo* transcriptome assembly for wild *Malus* accessions.

Parameter	*M. toringo*	*M. micromalus*
**Assembled transcripts**	139,716	132,885
**Ex90N50**	2,636	2,585
**Unigenes**	104,138	97,838
**ORFs**	38,730	36,443
**Completeness**	90.0%	90.0%
**Orthologues with *M.*** × ***domestica***	13,860	13,734

### *In silico* analysis and expression analysis by RT-qPCR

We selected genes potentially involved in the DHC pathway of wild *Malus* species for further expression analysis by RT-qPCR. We found a gene in *M. toringo* and *M. micromalus* that was up-regulated 8.6- to 11.3-fold compared to domesticated apple (**Supplementary Table 5**), annotated as phloretin 4′-*O*-glycosyltransferase. This gene resulted to encode for PGT2, a glycosyltransferase recently characterised to be involved in trilobatin biosynthesis (Wang et al., 2020). We included *CHS*, and *PAL1* to the expression analysis, considering their role in both flavonoid and DHC pathway (Ibdah et al., 2018). In addition, two genes of *M. toringo* and *M. micromalus* were selected as potential 3-hydroxylases to be further evaluated: ‘TRINITY_DN55953_c0_g1_i1.p1’ and ‘TRINITY_DN11639_c0_g1_i2.p1’, annotated as flavonol synthase/flavanone 3-hydroxylase-like. In addition, ‘TRINITY_DN56_c0_g1_i4.p1’ and ‘TRINITY_DN274_c0_g1_i3.p1’ were annotated as cytochrome P450 98A2, and were 36.7- to 36.8-fold up-regulated in *M. toringo* and *M. micromalus* compared to the orthologous gene of domesticated apple (MD08G1242900). A second gene in apple genome (MD08G1243000) has a predicted annotation as cytochrome P450 98A2, although it was not identified as the orthologue for ‘TRINITY_DN56_c0_g1_i4.p1’ and ‘TRINITY_DN274_c0_g1_i3.p1’ according to RBH. Among the first 5 best BLAST hits, we found a *p*-coumaroyl-shikimate 3′-hydroxylase from *P.* × *bretschneideri* (99% query cover and 93.36% identity). Cytochrome P450 genes were classified into CYP98 subfamily of cytochrome P450 enzymes (Nelson, 2009) and were assigned the following names: CYP98A195 and CYP98A196 from *M. toringo* and *M. micromalus*, respectively; and CYP98A197 (MD08G1242900) and CYP98A198 (MD08G1243000) from *M.* × *domestica*. Since cytochrome P450-dependent monooxygenases flavonoid 3′-hydroxylase (F3′H) and chalcone 3-hydroxylase (C3H) are capable of introducing hydroxyl groups in the B ring of flavonoids and DHCs, we aligned the amino acid sequences of the candidate genes of wild *Malus* with known C3H, F3′H, F6H and F3′5′H of other species and built a phylogenetic tree (**Supplementary Figure 4**). Proteins of *M. toringo* and *M. micromalus* clustered together, apart from F6H, F3′5′H and F3′H groups (**Supplementary Figure 4A**), suggesting that these genes might diverge in function. The closest members to CYP98A proteins of *Malus* wild species are CYP98A21 and CYP98A6 from *Ammi majus* and *Lithospermum erythrorhizon*, respectively (**Supplementary Figure 4B**). As a first approach of better characterising these candidate genes, we evaluated their expression profiles by RT-qPCR in selected tissues. The highest expression level of *F3′H-like* was found at fruit set of *M. toringo* and *M. micromalus*, followed by bloom and pink flowers. Leaves showed the lowest expression levels in both wild accessions, although significantly higher compared to *M.* × *domestica* (**Figure 3A**). *CYP98A195-196* showed high expression levels in pink and bloom flowers, as well as in young leaves (**Figure 3B**). *CYP98A195-196* transcript was also detected in fruit set of wild species, although significantly lower expression was found compared to leaf and flower tissues (**Figure 3B**). We also tested a third gene annotated as geraniol 8-hydroxylase-like by RT-qPCR, which showed the highest expression levels in *M.* × *domestica* young leaves, fruit set, and flowers at bloom stage (**Figure 3C**). In pink flowers, geraniol 8-hydroxylase-like showed similar expression levels between *M. micromalus* and *M.* × *domestica*, with statistically lower expression in *M. toringo*. In addition, the maximum expression for wild accessions was found in fruit set, showing no statistical difference with fruit set of domesticated apple (**Figure 3C**). The high expression in domestic apple along with higher expression in fruit set compared to the tissues where sieboldin is predominantly accumulated were used as criteria to exclude this third putative hydroxylase for further analyses. Meanwhile, *PAL1* was expressed at similar extent in all tissues analysed (**Figure 3D**). Similarly, *CHS* was found in all accessions, showing its highest expression in young leaves, followed by pink and blooming flowers (**Figure 3E**). As in the case of *CYP98A195-196*, *CHS* was also expressed in fruit set, with ‘Golden Delicious’ exhibiting a significant higher relative expression compared to wild accessions. *PGT2* was highly expressed in *M. micromalus* in pink flowers and young leaves, whilst *M. toringo* showed significantly lower expression levels in flower and leaf tissues, although no statistical difference was found at fruit set of both accessions (**Figure 3F**).

### *De novo* production of sieboldin in *Saccharomyces cerevisiae*

In order to assess the 3-hydroxylase activity of candidate genes identified by the transcriptomic analysis of wild *Malus* accessions, we established a heterologous system (summarised in **Figure 4A**) in a phloretin-producing *S. cerevisiae* strain by expressing *PAL*, *C4H*, *4CL2*, *CHS* and *CPR* as previously described (Eichenberger et al., 2017). Candidate *F3′H-like* and *CYP98A195-196* genes of *M. toringo* and *M. micromalus* were cloned into a high copy yeast expression vector pAT425 and transformed in the phloretin-producing yeast strain. Since pAT425 vector harbours two cloning sites, under the regulation of two different constitutive promoters for yeast expression (Ishii et al., 2014), we initially tested 3-hydroxylase candidate genes under the control of alcohol dehydrogenase (ADH1) and glyceraldehyde-3-phosphate dehydrogenase (TDH3) promoters after 48 h of incubation. Interestingly, 3-hydroxyphloretin was detected only in the phloretin-producing strains expressing the genes *CYP98A195* and *CYP98A196* of *M. toringo* and *M. micromalus*, respectively (**Figure 4B**). Moreover, expression of *CYP98A195-196* under TDH3 promoter led consistently to higher levels of 3-hydroxyphloretin, compared to expression under ADH1 promoter (**Figure 4B**). To further evaluate the possibility of achieving *de novo* production of sieboldin in *S. cerevisiae* using *CYP98A196-197* of wild species, we cloned *CYP98A197-198* of *M.* × *domestica* into the same backbone and co-expressed each CYP98A gene of wild and domesticated *Malus* accession with the recently characterised 4′-*O*-glycosyltransferase (PGT2) involved in trilobatin synthesis, which also accepts 3-hydroxyphloretin as substrate *in vitro* (Wang et al., 2020). To this aim, we cloned *PGT2* under ADH1 promoter into the pAT425 vector harbouring the constructs pTDH3::CYP98A195, pTDH3::CYP98A196, pTDH3::CYP98A197 or pTDH3::CYP98A198, and transformed each vector in the phloretin-producing yeast strain followed by incubation for 72 to 96 h. Interestingly, expression of *CYP98A* genes from *M.* × *domestica* did not produce 3-hydroxyphloretin even after 96 h, whereas an increase in 3-hydroxyphloretin was shown in yeast expressing *CYP98A195* and *CYP98A196* from 72 to 96 h (**Figure 4D**, **Supplementary Figure 5**). Furthermore, sieboldin was produced only when *CYP98A* members from wild accessions were used (**Figure 4F**, **Supplementary Figure 5**), leading to levels ranging from 2 - 3 to 3 - 4.5 mg·L^-1^ after 72 and 96 h, respectively (**Figure 4F**). As expected, phloretin was accumulated in all yeast strains at similar levels, with the yeast strain transformed with the empty pAT425 vector showing the highest values (**Figure 4C**). Meanwhile, trilobatin was produced in yeast co-expressing *PGT2* with *CYP98A* genes of wild and domesticated accessions, although higher level of trilobatin was observed in yeast harbouring *CYP98A* genes of *M.* × *domestica*, compared to those expressing *CYP98A195* and *CYP98A196* (**Figure 4E**).

### Assessment of germplasm collection with HRM marker for DHC3H

**Figure 5:**
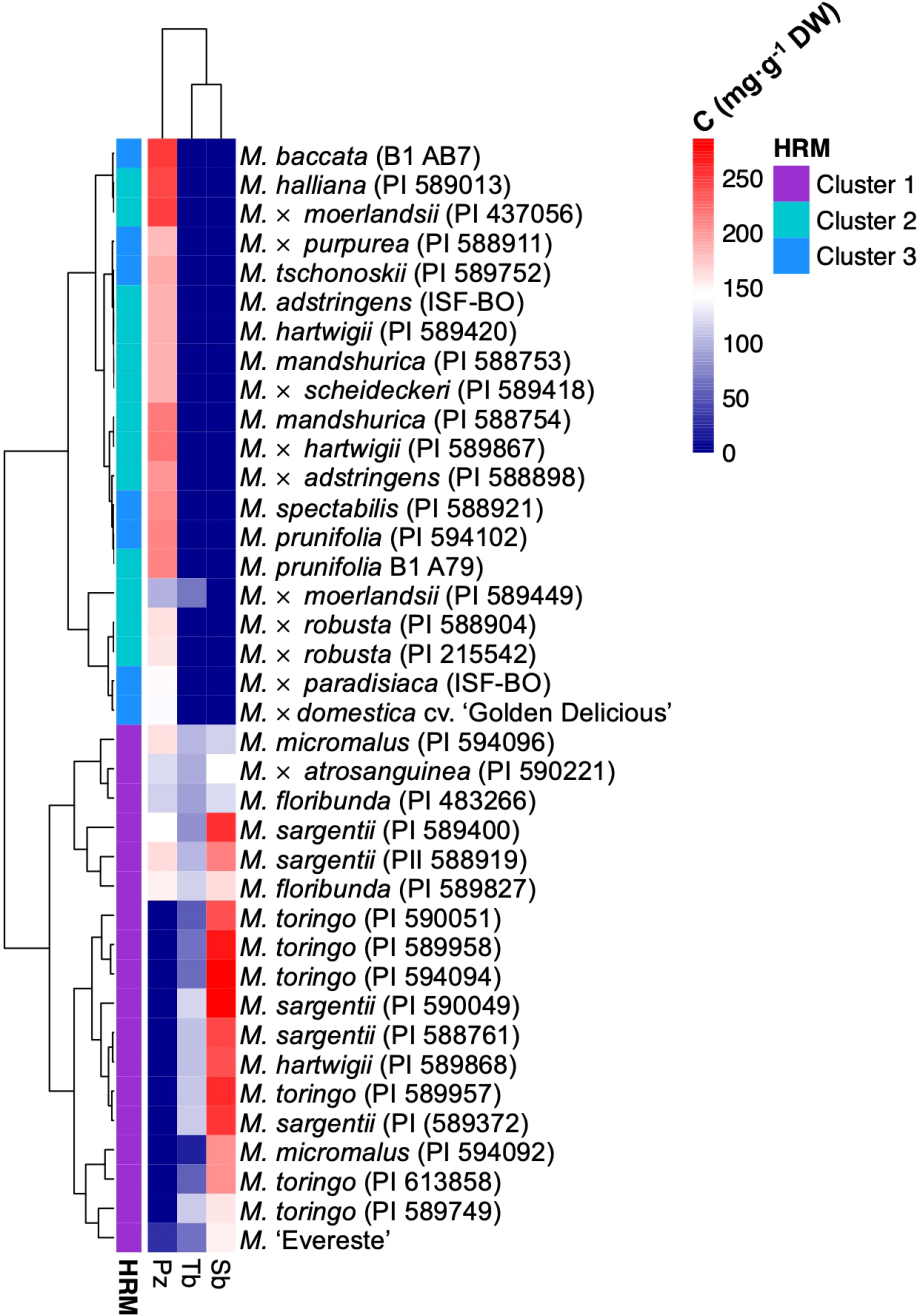
Heatmap clustering *Malus* germplasm collection by DHC profile and HRM marker analysis for *CYP98A195-198* isoforms. Hierarchical heatmap of the concentration of the main DHC forms phloridzin (Pz), trilobatin (Tb) and sieboldin (Sb), for wild and hybrid *Malus* accessions in a germplasm collection. Gradient of blue and red colours represent a trend of decrease and increase of metabolite, respectively. Metabolite clustering was automatically calculated for genotypes in R-environment. HRM clustering result is displayed at the left of each accession.

The orthologous gene of domesticated apple identified in RNA-Seq analysis and functionally characterised corresponds to MD08G1242900 and assigned the name CYP98A197. However, we also identified an adjacent gene in apple annotated as CYP98 A2 in chromosome 8 of the reference ‘Golden Delicious’ genome, corresponding to the gene MD08G1243000 and named CYP98A198. This second gene of domesticated apple shares 95.7% and 93.4% nucleotide and amino acid identity with wild accessions, respectively. Aiming to determine whether SNPs in *CYP98A* sequences could correlate with the presence or absence of sieboldin, we conducted genetic mapping by high resolution melting (HRM) marker analysis. Using primers flanking a region close to the start codon showing the highest sequence divergence among CYP98A195-198 sequences of wild and domesticated species (**Supplementary Figure 6A**), HRM marker analysis identified three different clusters in the *Malus* germplasm accessions with an average confidence level of 98.1% (**Supplementary Figures 6B, C**, **Supplementary Table 7**). The three glycosylated DHCs derived from phloretin (phloridzin, trilobatin and sieboldin) found in *Malus* spp. were determined in young leaves of each genotype. Interestingly, the genotypes evaluated that accumulate sieboldin clustered in group 1 (including *M. sargentii*, *M. toringo*, *M. micromalus* and *M.* ‘*Evereste*’), whereas accessions accumulating exclusively phlorizin (including *M. baccata*, *M.* × *domestica*, *M. prunifolia*, *M. robusta*, *M. halliana*, *M.* × *paradisiaca*, *M.* × *moerlandsii*) categorised in clusters 2 or 3 (**Figure 5**). *M. floribunda* and *M.* × *atrosanguinea*, as well as some accessions of *M. micromalus* and *M. sargentii* accumulate both phloridzin and sieboldin, although they clustered together with accessions where only sieboldin is produced, and HRM marker analysis categorised them in cluster 1 (**Figure 5**).

### Characterisation of DHC3H protein by subcellular localization and protein modelling

**Figure 6:**
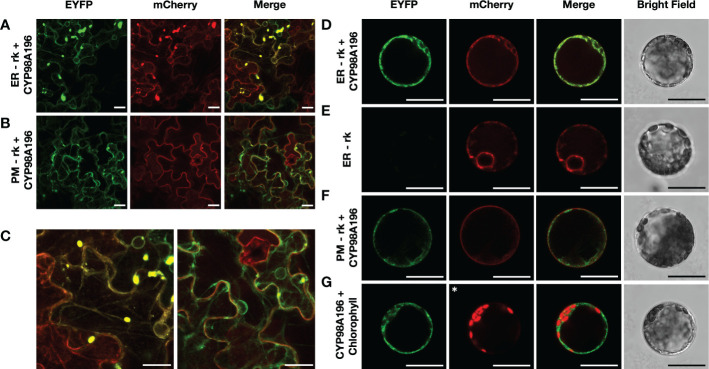
Determination of sub-cellular localisation of CYP98A196. Tobacco epidermal cells were transiently transformed with CYP98A196-EYFP construct by agroinfiltration in a stable transgenic tobacco line expressing the endoplasmic reticulum ER-rk marker **(A)** or the plasma membrane PM-rk marker **(B)**, both fused to mCherry. Representative results of each construct are presented for EYFP, mCherry and merged visualization, and a zoom of merged fields following co-infiltration of ER-rk and PM-rk with CYP98A196-EYFP are presented in **(C)**, left and right panel, respectively. In addition, protoplasts co-expressing ER-rk marker with CYP98A196-EYFP **(D)** or empty vector **(E)**, or PM-rk marker with CYP98A196 **(F)** are presented for EYFP, mCherry, bright field and merged visualization. **(G)** CYP98A196-EYFP and autofluorescence of chlorophyll instead of mCherry (indicated with an asterisk) collected as described in Materials and Methods. Scale bar: 25 µm.

Since several steps of the flavonoid pathway occur in the endoplasmic reticulum (Agati et al., 2012), we aimed to determine the subcellular localisation of CYP98A195-196 identified from wild *Malus* species. We established stable transgenic lines of tobacco overexpressing protein markers of endoplasmic reticulum and plasma membrane fused to mCherry, using ER-rk and PM-rk vectors (Nelson et al., 2007). We selected *CYP98A196*, given that genes from both wild accessions exhibit 100% amino acid identity. Transient expression of CYP98A196 fused to EYFP in tobacco leaves of transgenic ER-rk and PM-rk lines showed a distinctive pattern (**Figure 6A**, left and middle panel) that co-localised with the emission of ER-rk marker (**Figure 6A**, right panel). Fluorescence of CYP98A196-EYFP was distributed in the periphery of epidermal cells showing the characteristic pattern of a network close to the cell borders (**Figure 6C**, left panel), underneath a more homogeneous fluorescence signal along the surface of cells exhibited by the plasma membrane marker (**Figure 6B**, middle panel; **Figure 6C**, right panel). Protoplasts of tobacco leaves were isolated for the same constructs, and CYP98A196-EYFP showed a clear cell peripheral pattern with complete co-localization with ER-rk marker (**Figure 6D**), similar to the pattern displayed by the marker transformed with the empty vector (**Figure 6E**). The peripheral localisation of CYP98A196 does not co-localize with the plasma membrane signal, which surrounds the cell in a uniform manner (**Figure 6E**). The signal of CYP98A196-EYFP surrounds the autofluorescence of chlorophyll, as depicted in **Figure 6G**

**Figure 7:**
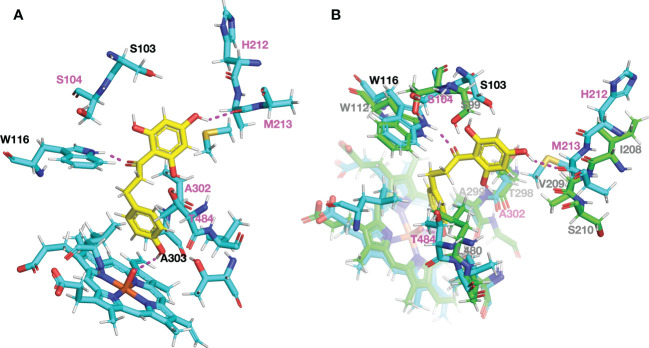
Structural comparison of the ligand pocket predicted for DHC3H model showing 3-hydroxylase activity *in vivo*. Protein models and docking with phloretin (yellow) were obtained using PCPD and PCPLD, respectively. **(A)** Amino acid residues found exclusively in CYP98A196 around phloretin within 5 Å are highlighted in magenta (S104, H212, M213, A302, T484), and predicted hydrogen bonds are indicated with dashed lines in magenta. **(B)** Superimposed models of CYP98A196 with AtCYP98A3, a characterised 3-hydroxylase. Carbon, oxygen, nitrogen and sulphur atoms are shown in light blue, red, blue and orange, respectively.

To examine the structural differences of CYP98A members producing 3-hydroxyphloretin *in vivo* to those of *M.* × *domestica*, we constructed protein models using PCPD server, which combines structural homology and molecular dynamics simulation optimised for plant cytochrome P450s to predict the final protein model (Wang et al., 2021). Then, we carried out a protein-ligand docking by PCPLD using phloretin as potential ligand to predict the position and orientation of phloretin in CYP98A196 protein model. Phloretin is predicted to interact by hydrogen bonds with the residues Trp116 and Met213 and with the heme group. Focusing the attention to residues in the proximity of the ligand pocket predicted, we found that most of the residues within 5 Å were conserved. Five residues, however, were exclusively found in CYP98A196 (Ser104, His212, Met213, Ala302, Thr484). Among them, His212 and Met213 are in close proximity to the A ring of phloretin, with Met213 forming a hydrogen bond with the hydroxyl group at 4′ position of A ring (**Figure 7A**). In addition, the polar residue Thr302 found in CYP98A197 of *M.* × *domestica* has the counterpart with the aliphatic Ala302 in CYP98A196 of *M. micromalus*, showing physico-chemical conservation with Val302 of CsC3H (**Supplementary Figure 7**), the only example with reported 3-hydroxylase activity producing 3-hydroxyphloridzin in *CsC3H*-overexpressing apple lines (Hutabarat et al., 2016). Superimposition of CYP98A196 with the protein model of a CYP98 member coding for C3H that exhibits 3-hydroxyalse activity towards coumaroylshikimate (Schoch et al., 2001) shows that Trp116 of CYP98A196 and Trp112 of C3H are close to the oxygen in the carbonyl group linking A and B ring of phloretin (**Figure 7B**).

## Discussion

### Detailed profiling of dihydrochalcones during plant development in *Malus* accessions

DHCs are a class of secondary metabolites of the phenylpropanoid pathway, which includes anthocyanins, flavonols and flavan-3-ols with reported benefits for human health (Panche et al., 2016). In contrast to the more common polyphenols, DHCs have a limited natural distribution and the major flavonoid class found in *Malus* spp. (Rivière, 2016). Phloridzin, the main DHC form of *M.* × *domestica* (Gosch et al., 2010b), was thought for long time to be exclusive to apple, leading to propose phloridzin as an authenticity marker for evaluation of apple-derived products in the food industry (Hrubá et al., 2021). Most of the studies have focused on phloridzin, given the protective effects of phloridzin reported in mammal models (Stangl et al., 2005; Szliszka et al., 2010; Viet et al., 2013). However, trilobatin and sieboldin are accumulated in considerable amounts in other *Malus* species (Gutierrez et al., 2018b). Despite the properties of DHCs as flavour sweeteners and bitterness blockers (Tomás-Barberán et al., 1995; Wang et al., 2020), studies addressing the accumulation patterns and physiological changes of DHCs *in planta* are still scarce. Histolocalisation showed that DHCs are present in proximity to the vascular system and upper layers of leaves in the ornamental *M.* cv. ‘*Evereste*’ (Gaucher et al., 2013). Phloridzin is found in bark (Zhang et al., 2007), root exudates (Hofmann et al., 2009) and leaves of domesticated apple (Petkovsek et al., 2009). In addition, apple cultivars accumulate varying amounts of phloridzin in skin and whole fruit (Łata et al., 2009). Here, we conducted a detailed profiling of DHCs during bud and fruit development, along with a systematic monitoring of foliar DHC content. Polyphenolic profiles of the wild accessions *M. toringo* and *M. micromalus* were dominated by the glycosylated DHCs sieboldin, followed by trilobatin, whereas domesticated apple only accumulates phloridzin (**Figure 1**). Aglycones phloretin and 3-hydroxyphloretin were detected in low concentrations in leaves and fruit set, which may suggest that glycosylation stabilises phloretin and 3-hydroxyphloretin as previously hypothesised (Caputi et al., 2012; Ibdah et al., 2018). We observed a drastic increase of phloridzin, trilobatin and sieboldin in half-inch buds after dormancy (**Figures 1A, D, G**), reinforcing a previous study that proposed phloridzin as a phase change in apple (Zhang et al., 2007). Foliar content of DHCs was relatively stable across the growing season, showing higher levels of trilobatin in *M. micromalus* compared to *M. toringo* (**Figures 1D, E**), although sieboldin was the predominant DHC found in leaves and exhibited similar levels in both wild accessions (**Figure 1H**). A previous study showed that sieboldin exerted the highest antioxidant capacity in young leaves, suggesting a role in coping with oxidative stress during development (Dugé de Bernonville et al., 2010). Fruit of wild accessions showed low DHC content compared to leaves and buds, which has been attributed to a dilution effect in domesticated cultivars caused by fruit size (Busatto et al., 2019). In agreement with this hypothesis, we found the highest concentration of phloridzin, trilobatin and sieboldin at fruit set, which constantly decreased until fruit ripening (**Figure 1**, **Supplementary Figure 3**). Most of the DHC content in wild and domesticated accessions was attributed to the peel, which in turn has shown positive correlation between DHCs levels in peel and antioxidant capacity (Zhou et al., 2018), further suggesting a role of flavonoids and, among them, DHCs in the response of *Malus* spp. to oxidative stress faced during the growing season (Tsao et al., 2005).

### Identification of candidate genes involved in sieboldin biosynthesis by *de novo* transcriptome assembly and RNA-Seq analysis

Public genetic resources in *Malus* species have increased in the last two decades, from the reference genomes for *M.* × *domestica* cv. ‘Golden Delicious’ (Velasco et al., 2010; Daccord et al., 2017) and ‘Gala’ (Sun et al., 2020) to sequencing of ‘Gala’ wild progenitors *M. sieversii* and *M. sylvestris* (Sun et al., 2020), as well as wild *M. baccata* and *M. prunifolia* (Chen et al., 2019; Li et al., 2022). Wild accessions with publicly available genome resources, however, accumulate exclusively (*M. sieversii*, *M. sylvestris* and *M. baccata*) or predominantly (*M. prunifolia*) phloridzin according to a previous study (Gutierrez et al., 2018b). Therefore, we chose two *Malus* accessions from the germplasm collection at Fondazione Edmund Mach accumulating sieboldin and trilobatin to initially conduct an RNA-Seq analysis. As a preliminary approach to identify genes that could be involved in sieboldin biosynthesis, we hypothesised that candidate genes of wild accessions should be highly up-regulated compared to the domesticated apple reference genome, provided that reads of wild species align in similar proportion to apple genome. We found that the percentage of mapping was comparable in all genotypes (72.7% in Golden Delicious, 71.5 – 73% for *M. toringo* and *M. micromalus*; **Supplementary Table 4**), indicating that our preliminary approach led to mapping statistics within the acceptable range when conducting RNA-Seq analysis (Conesa et al., 2016). Single-end reads tend to have a slightly lower percentage of uniquely mapped reads (Chhangawala et al., 2015), which may explain the mapping percentage in all genotypes. In addition, multiple mapping was similar across accessions (**Supplementary Table 4**) that could be explained by recent duplication events during domestication of apple (Velasco et al., 2010; Daccord et al., 2017). Importantly, we identified two candidate genes: *F3′H-like*, annotated as flavonol synthase/flavanone 3-hydroxylase; and *CYP98A195-196*, that had also annotation as *p*-coumaroyl-shikimate 3′-hydroxylase. These genes showed a 4.3 – 4.9-fold (*F3′H-like*) and 36.8-fold (*CYP98A195-196*) up-regulation in *M. toringo* and *M. micromalus*, respectively (**Supplementary Table 5**). Cytochrome P450-dependent monooxygenases F3′H and F3′5′H can also introduce hydroxyl groups at the 3′ or 3′ and 5′ position of the B ring of flavonoids, showing differences in substrate specificity and amino acid identity lower than 50% (Seitz et al., 2006). We found that the two candidate genes of wild *Malus* species clustered (CYP98A195-196) in a phylogenetic tree apart from known F3′H, F3′5′H and F6Hs. To delve into the potential candidates of wild accessions for expression and functional analyses, we did a *de novo* transcriptome assembly from young leaves, resulting in around 100,000 unigenes and completeness of 90%. These results are in line with previous *de novo* transcriptomes assembled in other species (Wu et al., 2017; Biswal et al., 2021; Madritsch et al., 2021; Pacheco et al., 2021), where a great proportion of assemblies are short or partial transcripts, as well as pre-mRNA, ncRNA and transcript artifacts (Raghavan et al., 2022). To compensate for the low-expressed and short transcripts that dominate *de novo* transcriptomes, it has been proposed that expression-informed statistics such as Ex90N50 may reflect better the length of assembled transcripts accounting for 90% of the normalised expression data (Hölzer and Marz, 2019). In our study, Ex90N50 values for *M. toringo* and *M. micromalus* were around 2.6 kb, suggesting that half of the transcripts assembled accounting for 90% of the expression data had an acceptable size to retrieve full-length CDS. Prediction of ORFs by TransDecoder resulted in around 36,000 – 39,000 predicted CDS, close to the genes of *M.* × *domestica* (Daccord et al., 2017). In addition, we identified several genes involved in the flavonoid pathway, including *PAL1*, *CHS* and *PGT2*. To validate transcriptomic results, we conducted an expression analysis of putative 3-hydroxylases together with genes involved in the phenylpropanoid pathway in selected tissues being the predominant sources of DHCs according to the metabolite profiling. We confirmed that the expression of *PAL1* and *CHS* pattern was similar for all accessions (**Figures 3D, E**), suggesting that the key steps of the phenylpropanoid pathway are transcriptionally active throughout the developmental stages evaluated. *PGT2* expression was detected only in wild accessions, showing its maximum in young leaves and pink flowers of *M. micromalus* (**Figure 3F**). Moreover, we analysed three potential 3-hydroxylase candidates, and selected *F3′H-like* and *CYP98A195-196* genes for further analyses, using as criteria the high expression in wild accessions compared to domesticated apple and expression in tissues where DHCs are mainly accumulated.

### Functional characterisation of 3-hydroxylase involved in the production of sieboldin *in vivo*

Several examples utilise *de novo* transcriptome assemblies for identification of putative genes involved in the naturally-abundant flavonoids (Thole et al., 2019; Biswal et al., 2021), carotenoids (Pacheco et al., 2021) or glucosinolates (Wu et al., 2017). These studies have focused their attention on validating *de novo* transcriptomes by RT-qPCR to evaluate transcriptional regulation of the genes of interest. To the best of our knowledge, the use of high throughput platforms to attempt the elucidation of genes involved in specialised metabolism of non-model species is still scarce in literature, which counts with examples of GWAS and RNA-Seq in the case of DHCs of apple (Zhou et al., 2017; Kumar et al., 2022). Aiming to provide functional evidence for the involvement of candidate genes in the biosynthesis of the specialised metabolites 3-hydroxyphloretin and sieboldin, we used *S. cerevisiae* as an heterologous system to test the *in vivo* activity of candidate P450 monooxygenases, which is a readily suitable model for expressing P450 monooxygenases, because of its endogenous NADPH-cytochrome P450 reductases (CPR) needed for the activity of P450 monooxygenases (Hausjell et al., 2018). Using a yeast strain producing phloretin by metabolic engineering as stablished before (Eichenberger et al., 2017), we expressed candidate genes under the control of two constitutive promoters for protein expression (ADH1, TDH3). Interestingly, we found that only CYP98A195 and CYP98196 were able to convert phloretin into 3-hydroxyphloretin (**Figure 4**) independently of the promoter used, indicating that CYP98A proteins from wild *Malus* species exerts a 3-hydroxylase activity *in vivo*. Phloretin conversion was higher under TDH3 promoter, which is consistent with the higher GFP signal obtained under the control of TDH3 compared to ADH1 promoter (Ishii et al., 2014). We therefore chose the expression under TDH3 promoter to further assess the production of sieboldin by introduction of *PGT2*, a 4′-*O-*glycosyltransferase that accepts 3-hydroxyphloretin *in vitro* (Wang et al., 2020). We demonstrated that *CYP98A195* and *CYP98A196* co-expressed with *PGT2* in the phloretin-producing yeast can convert 3-hydroxyphloretin to its glycosylated form sieboldin, which was not found with the apple proteins of *M.* × *domestica*, even after 96 h of incubation (**Figure 4**). Moreover, we aimed to develop a HRM marker to discriminate different alleles, considering the nucleotide differences found between *CYP98A195-196* of wild accessions and *M.* × *domestica CYP98A197-198*. Screening a *Malus* germplasm collection by HRM marker analysis, we recognised three clusters, which are consistent with the nucleotide differences found between *CYP98A* sequence of *M. toringo* and *M. micromalus* and the two genes of *M.* × *domestica* (**Supplementary Figure 6**, **Supplementary Table 7**). Furthermore, these clusters correlated with the presence or absence of sieboldin (**Figure 5**), indicating that *CYP98A* isoforms may be useful to recognise sieboldin-producing *Malus* accessions. A previous study categorised *Malus* accessions by the presence of only phloridzin (P), phloridzin and trilobatin (PT), sieboldin and trilobatin (ST) or the three glycosylated DHCs (SPT) (Gutierrez et al., 2018b). In agreement with the accessions of the USDA Geneva *Malus* Core collection, accessions of *M. toringo* and *M. sargentii* of our germplasm collection displayed ST and SPT profiles, respectively, and HRM analysis detected the allele present in *M. toringo* and *M. micromalus*. It has been proposed that sieboldin originated from *M. toringo*, and hybrids of *M. toringo* with phloridzin-accumulating accessions (*M. baccata*, *M. halliana*, *M. prunifolia*) derives in accessions with ST and SPT profiles (Gutierrez et al., 2018b). The latter progenitors belonged to clusters 2 or 3, meaning that they showed a HRM profile similar to the inactive CYP98A197 and CYP98A198 genes of *M.* × *domestica*. Hybrids of *M. toringo* with phloridzin-accumulating parental accessions evaluated in the germplasm collection (*M. floribunda* and *M.* × *atrosanguinea*) exhibited a SPT profile. Despite the intermediate levels of the three DHCs compared to accessions with P and PT profiles, HRM analysis identified the allele of *M. toringo* and *M. micromalus*. Altogether, these results indicate that differences in CYP98A members may be used for screening *Malus* accessions accumulating exclusively sieboldin or together with other DHCs. A linkage and association analysis of DHCs identified LG7 and LG8 as major determinant loci for trilobatin and sieboldin (Gutierrez et al., 2018a). PGT2 mapped in LG7 of *M.* × *domestica* genetic map, corresponding to gene models MD07G1281000 and MD07G1281100 in apple found in chromosome 7 (Wang et al., 2020). Similarly, the genes identified in *M. toringo* and *M. micromalus* in our study have their equivalents in chromosome 8 of apple (MD08G1242900 and MD08G1243000), suggesting that *CYP98A* allele of wild *Malus* accessions may explain the QTL associated with sieboldin identified in LG8 in Gutierrez et al., 2018a.

To further characterise CYP98A proteins of wild *Malus* species, we selected CYP98A196 because the amino acid sequence has 100% amino acid identity with CYP98A195 despite sharing 99.94% nucleotide identity, indicating that nucleotide differences correspond to synonymous mutations. It has been proposed that the phenylpropanoid pathway exhibits subcellular organisation, where cytosolic proteins (including PAL and CHS) are associated with other proteins of the pathway anchored to the endoplasmic reticulum (such as C4H and F3′H), forming multienzyme complexes channelling intermediate substrates that compete at different branch points (Winkel-Shirley, 2001). Thus, we assessed the sub-cellular localisation of CYP98A196, and found that it exhibited the characteristic pattern of network around a structure compatible with nucleus. Moreover, CYP98A196 fused to EYFP co-localised with an ER marker created by combining the signal peptide of AtWAK2 at N-terminus and the HDEL ER retention signal at C-terminus of the fluorescent protein (Nelson et al., 2007). The peripheral pattern of CYP98A196-EYFP fusion protein did not co-localised with the plasma membrane marker, surrounding chloroplasts of epidermal cells of tobacco (**Figure 6**).

CYP98A proteins of wild *Malus* accessions exhibiting 3-hydroxylase activity share 93.36% - 93.5% amino acid identity with CYP98A197 and CYP98A198 of *M.* × *domestica*. Remarkably, these differences are sufficient to produce proteins lacking 3-hydroxylase activity towards phloretin in yeast. Aiming to explore the structural basis that could underlie differences in activity, we conducted a protein model prediction by PCPD, a plant cytochrome P450 database that combines AlphaFold2, molecular dynamics (MD) simulation and ligand docking to score and determine the final model (Wang et al., 2021). AlphaFold has greatly improved the accuracy of protein structure prediction, outperforming standard algorithms (Jumper et al., 2021). Integration of MD-simulation and ligand docking process optimised for cytochrome P450s (PCPLD) resulted in the construction of a broad database specifically designed for simulation of plant cytochrome P450s (Wang et al., 2021). Using PCPD and PCPLD, we found significant differences in DHC3H compared to *M.* × *domestica*. Six substrate recognition sites (SRS) have been described in cytochrome P450s (Gotoh, 1992): SRS1 (residues 103 - 126), SRS2 (residues 209 - 216), SRS3 (residues 248 - 255), SRS4 (residues 302 - 320), SRS5 (residues 375 - 385) and SRS6 (residues 485 - 493). Comparing the residues close to the predicted substrate phloretin between DHC3H and *M.* × *domestica*, we found that four mutations were located precisely in the substrate recognition sites (Ser104, SRS1; His212, SRS2; Met213, SRS3; Ala302, SRS4), whilst Thr484 was next to the SRS6 (**Figure 7**, **Supplementary Figure 7**). Moreover, two hydrogen bonds stabilise the interaction between A ring of phloretin and residues in SRS1 (Trp116, Met216). The former study characterising C3H demonstrated that changes in amino acid residues of SRS1 have an impact on chalcone acceptance (Schlangen et al., 2010). In addition, a previous work demonstrated that substrate specificity and functional differences between F3′H and F3′5′H are determined near the N-terminal (SRS1 - 3) and C-terminal (SRS6) regions, respectively (Seitz et al., 2007). Amongst the vast cytochrome P450 superfamily, nomenclature rules have been stablished to distinguish families (indicated by a number, sharing at least 40% amino acid identity), subfamilies (referred as letters, with at least 55% identity) and isoforms (gene identifiers) based on percentage of amino acid identity (Nelson, 2006). Importantly, several plant cytochrome P450s families have been implicated in the metabolism of specialised metabolites, with reported examples in cyanogenic glucosides, glucosinolates, terpenoids and phenylpropanoids (Kahn et al., 1999; Hamberger and Bak, 2013; Romsuk et al., 2022). Besides CYP71 clan displaying monoterpenoid and sesquiterpenoid diversity and CYP79 members producing amino acid-derived specialised compounds, CYP98A subfamily has been described to transition from general to specialised phenylpropanoid metabolism (Hamberger and Bak, 2013). CYP98A3 and CYP98A22 from *Arabidopsis thaliana* and *Ruta graveolens* have been implicated in the 3′-hydroxylation involved in biosynthesis of chlorogenic acid and 3-hydroxylation of lignin monomers (Schoch et al., 2001; Karamat et al., 2012). In addition, CYP98A14 of *Coleus blumei* is responsible for the 3- and 3′-hydroxylation involved in the biosynthesis of rosmarinic acid (Eberle et al., 2009). Within CYP98A subfamily, a study found that CYP98A family members are highly duplicated, accumulating mutations that have led some members to acquire subsidiary activities involved in the hydroxylation of *p-*coumaroyltyramine (Morant et al., 2007). Interestingly, gene duplication in the CYP98A8 and CYP98A9 members in *Arabidopsis* resulted in neofunctionalisation of each member for different substrates, driven by a positive-selective pressure for the acquisition of regulatory elements in the promoter region, transcriptional reprogramming and local nonsynonymous amino acid substitutions (Matsuno et al., 2009). It is likely that amino acid differences in CYP98A195-198 found in *Malus* species have undergone similar mutations leading to modifying enzymatic activity.

The high expression of *DHC3H* in the tissues where DHCs are mainly accumulated along with key amino acid features close to the substrate pocket predicted in the protein models of wild accessions may explain the *in vivo* conversion of phloretin into 3-hydroxyphloretin, which ultimately leads to sieboldin production by co-expression with *PGT2*. Domesticated apple lines overexpressing *CsC3H* resulted in the production of 3-hydroxyphloridzin, which correlated with reduced susceptibility to apple scab and fire blight, suggesting a beneficial role of 3-hydroxylated DHCs (Hutabarat et al., 2016). A recent study attempted to discover genes involved in this key step of sieboldin biosynthesis, although the genes *F3′HI* and *F3′HII* cloned and characterised from *M.* × *domestica* genome did not accept phloretin, leading the authors to conclude that F3′Hs identified were unlikely involved in the 3-hydroxylation towards sieboldin biosynthesis (Weissensteiner et al., 2021). F3′HI and F3′HII, however, accepted naringenin, dihydrokaempferol and kaempferol, indicating a role in the well-studied 3′-hydroxylation of the B ring of flavonoids. The F3′H candidate cloned from wild *Malus* accessions in our study might share this role, which could explain the lack of 3-hydroxylase activity towards phloretin in *S. cerevisiae*. Our study describes for the first time an *in vivo* 3-hydroxylase activity of genes characterised from wild *Malus* genotypes that accumulate sieboldin as main DHC.

## Conclusions

DHCs are specialised secondary metabolites that possess beneficial effects in human health, recently implicated in abiotic and biotic response of plants, as well as plant development. The biosynthesis and physiological regulation of DHCs, however, remains an active field of research. Most of the research has focused on discovering genes involved in phloridzin biosynthesis, the main DHC compound found in *M.* × *domestica*. Nevertheless, trilobatin and sieboldin are DHCs that dominate the phenolic profile of some *Malus* species. Aiming to gain a better understanding of the accumulation of DHCs and their physiological roles in *Malus*, our study contributes to elucidate the biosynthetic pathway by combining transcriptomic, metabolite and functional analyses. The key 3-hydroxylation converting the precursor phloretin into 3-hydroxyphloretin is the missing step that ultimately leads to sieboldin biosynthesis. Here, we selected candidate genes by RNA-Seq and *de novo* transcriptome assembly that were functionally assessed by metabolic engineering of *S. cerevisiae*. CYP98A195-196 from *M. toringo* and *M. micromalus* exerted 3-hydroxylase activity in yeast and proved to be an endoplasmic reticulum resident protein. Moreover, protein modelling and ligand docking evidenced key mutations that could explain the acceptance of phloretin to produce 3-hydroxyphloretin, which could be further assessed by site-directed mutagenesis in future studies. Importantly, this study identified genes from wild *Malus* relatives to domesticated apple that can lead to sieboldin accumulation *in vivo*, by co-expression with the 4′-*O*-glycosyltransferase *PGT2*. In addition, we developed an HRM marker analysis that correlated with the accumulation of sieboldin in wild and hybrid *Malus* accessions that could be used for selecting plant *Malus* material. Altogether, our work constitutes the first study reporting a key gene that could account for the missing 3-hydroxylation step involved in the biosynthesis of sieboldin, thus providing the basis for future establishment of genetic engineered lines assessing the physiological roles of this gene in plants.

## Data availability statement

The data presented in the study are deposited in the NCBI GenBank repository, accession numbers OP616740 to OP616743, and Sequence Read Archive (SRA) repository, BioProject number PRJNA890040.

## Author contributions

SMi and SMa conceived the idea and designed the experiments. AD and RE contributed to the discussion and experiment planning. SMi, SMa, and MM collected plant material. SMi carried out RNA-Seq, gene expression analysis and metabolic profiling. SMi and A-SK carried out yeast *in vivo* and *in vitro* assays. AA contributed to LC-MS/MS analysis and visualisation of results. A-SK and SMi were responsible for HRM marker analysis. SMi and JL conducted the *de novo* transcriptome assembly and functional annotation, as well as agroinfiltration, protoplast preparation and confocal imaging. SMi and SMa wrote the manuscript, with input from all authors. All authors contributed to the article and approved the submitted version.
