# Supplementary figures and images for: De novo transcriptome assembly and functional analysis reveal a dihydrochalcone 3-hydroxylase(DHC3H) of wild Malus species that produces sieboldin in vivo
Source: Front Plant Sci. 2022 Dec 16;13:1072765. doi: 10.3389/fpls.2022.1072765 (PMC9800874; doi:10.3389/fpls.2022.1072765)

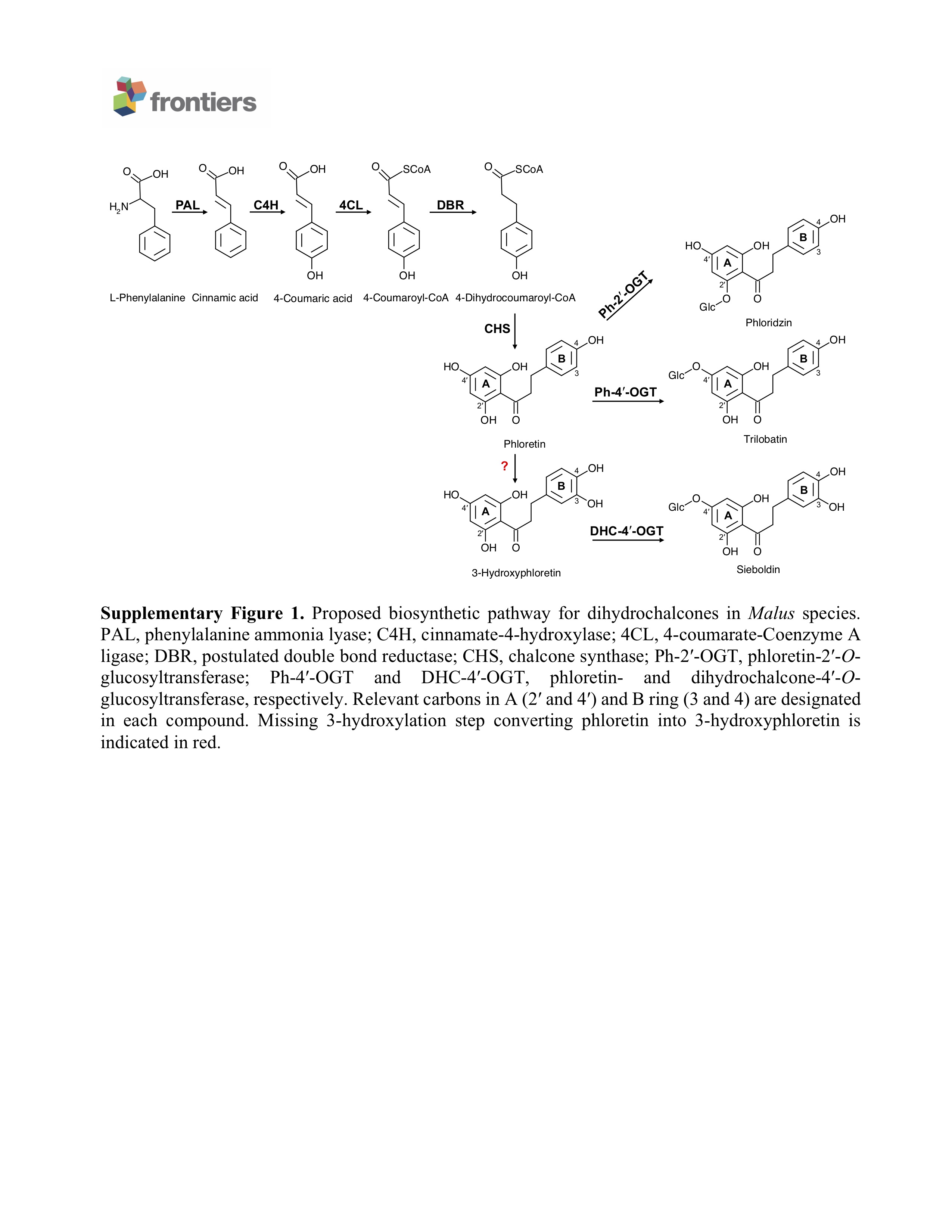

Supplement: Supplementary file 1 [file Image_1.jpg]

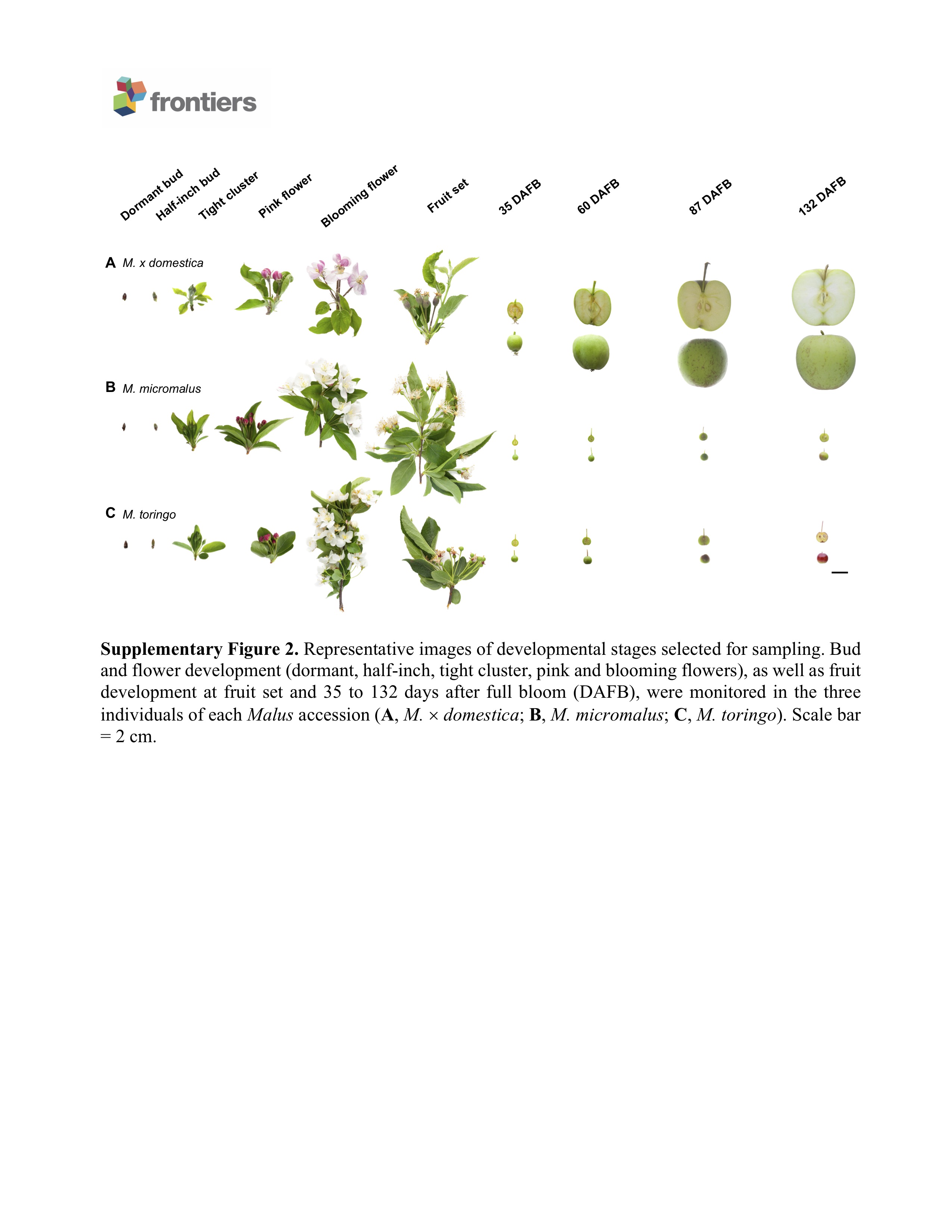

Supplement: Supplementary file 2 [file Image_2.jpg]

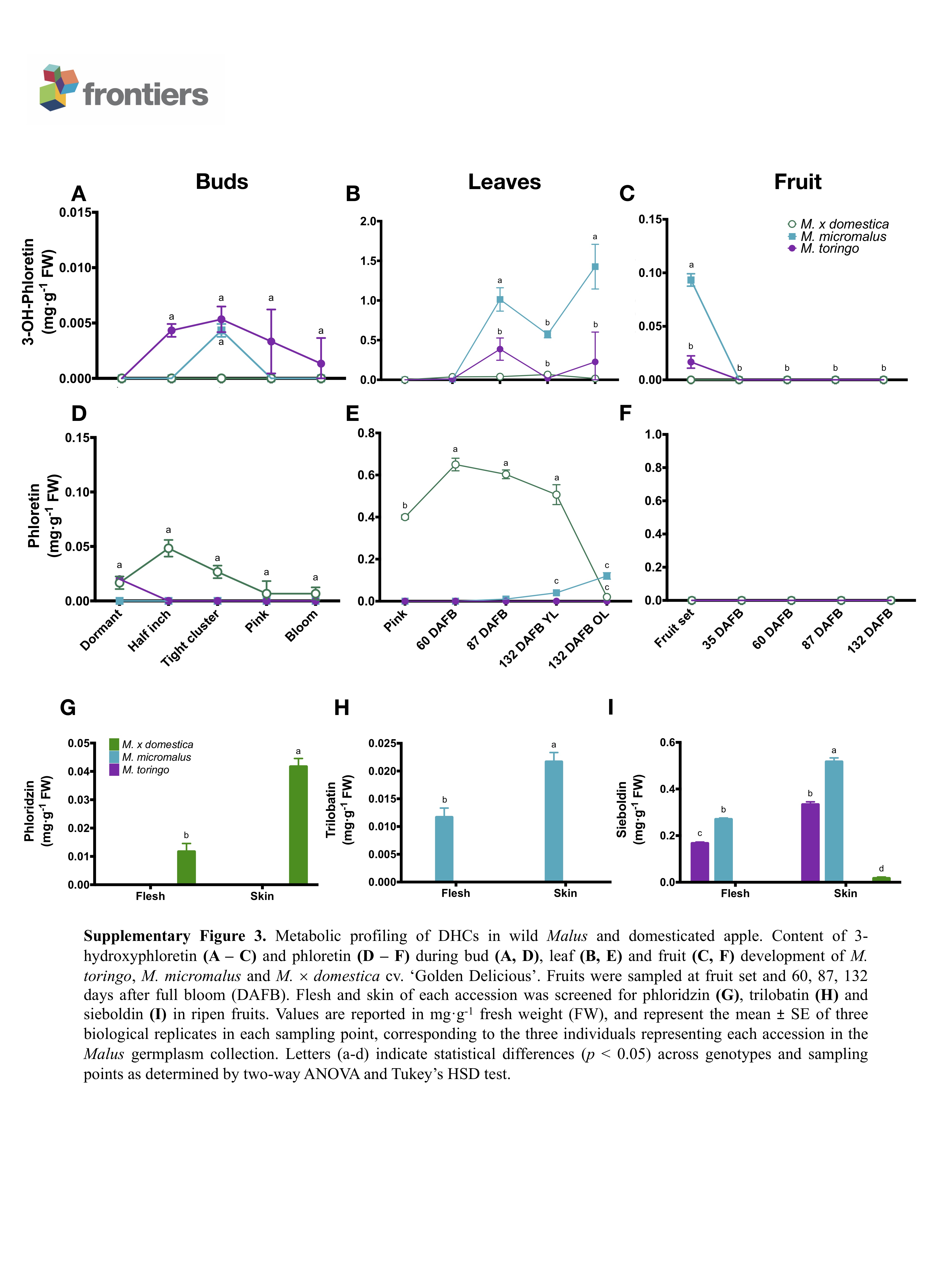

Supplement: Supplementary file 3 [file Image_3.jpg]

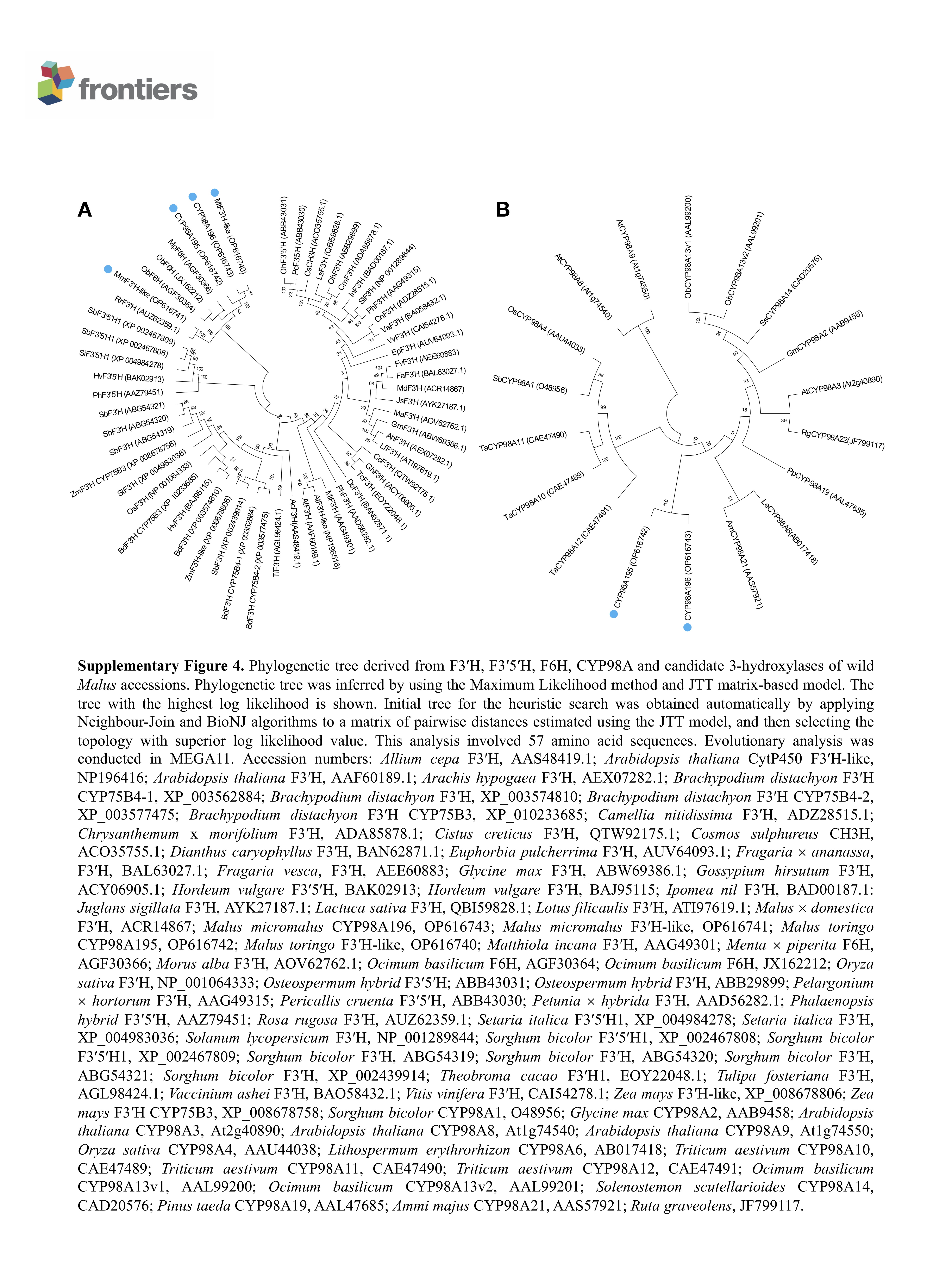

Supplement: Supplementary file 4 [file Image_4.jpg]

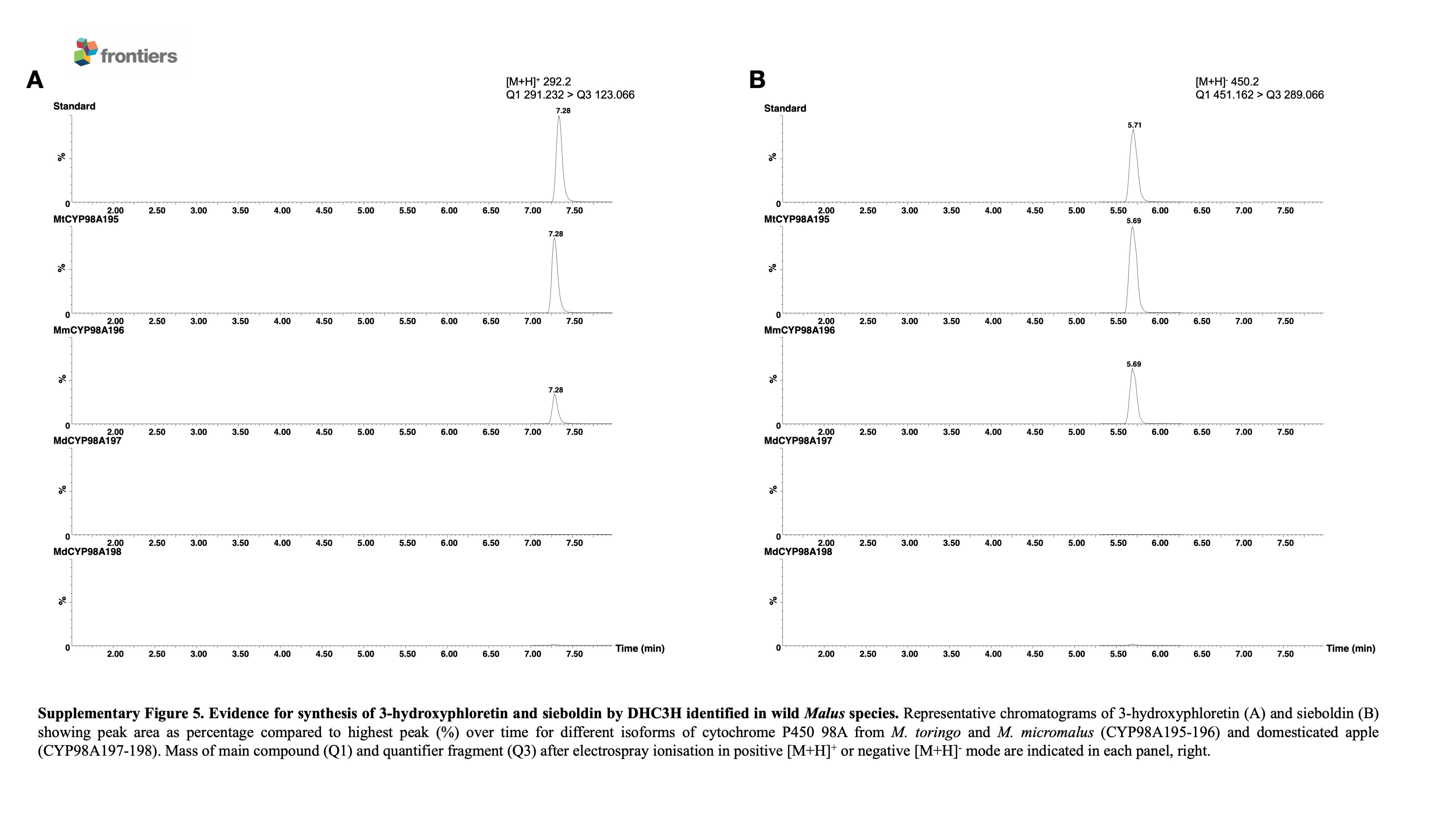

Supplement: Supplementary file 5 [file Image_5.jpg]

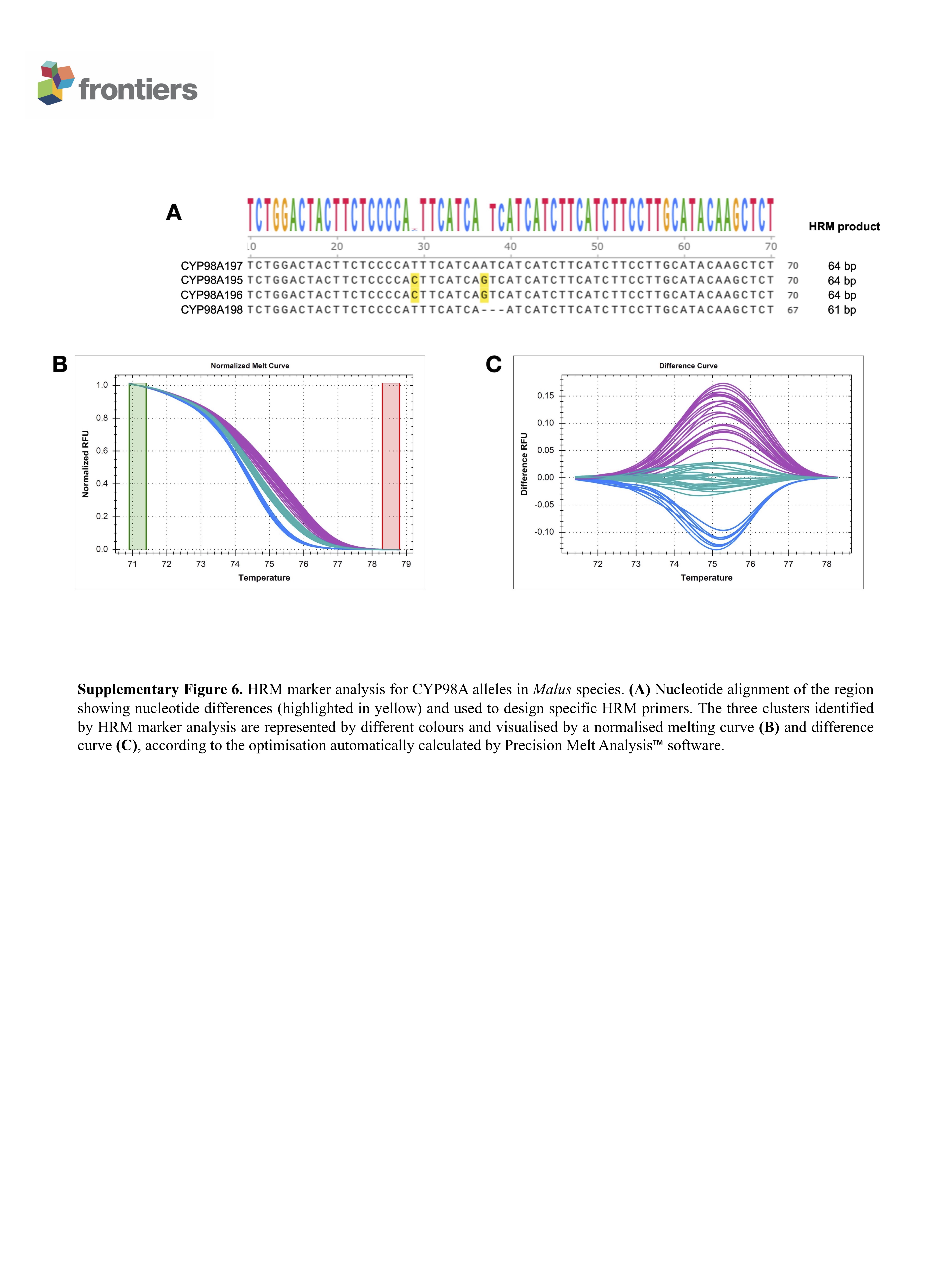

Supplement: Supplementary file 6 [file Image_6.jpg]

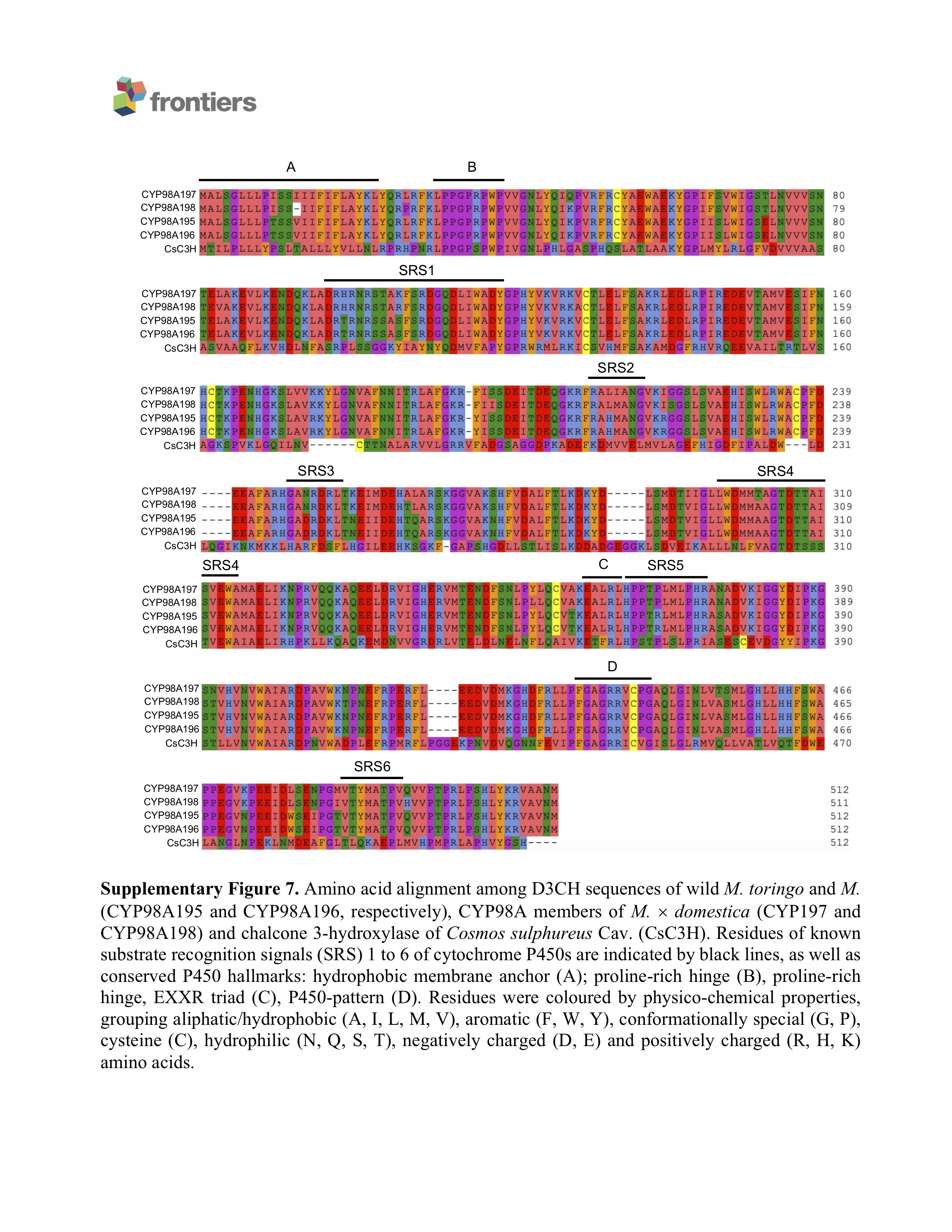

Supplement: Supplementary file 7 [file Image_7.jpg]
